# All-component-active metal–organic frameworks for tailored chemoradiotherapy of self-defensive tumors†

**DOI:** 10.1039/d5sc02482j

**Published:** 2025-06-26

**Authors:** Xiang Xu, Zhou Yang, Songsong Bao, Zhiyuan Xu, Tianrui Liu, Lina Wu, Jianping Lei

**Affiliations:** a State Key Laboratory of Analytical Chemistry for Life Science, State Key Laboratory of Coordination Chemistry, School of Chemistry and Chemical Engineering, Nanjing University Nanjing 210023 China jpl@nju.edu.cn; b State Key Laboratory of Microbial Technology, School of Food Science and Pharmaceutical Engineering, Nanjing Normal University Nanjing 210023 China wuln@njnu.edu.cn

## Abstract

Radiotherapy is a widely used clinical treatment for locoregional cancers, but it still faces radiation resistance arising from abundant glutathione (GSH) and DNA damage repair (DDR). To overcome these self-defense pathways, various radiosensitizers have often been integrated with pharmaceutical agents, forming hybridized carriers for combination therapy. Herein, an all-component-active metal–organic framework (aaMOF), composed of chemotherapeutic thioguanine as a linker and copper iodide as nodes, is rationally designed for tailored chemoradiotherapy against tumor self-defense pathways. Unlike conventional carrier-based systems, aaMOF releases all active components (copper iodide and thioguanine) upon GSH-triggered disassembly. Subsequently, high levels of DNA double-stranded breaks and reactive oxygen species (ROS) can be generated by iodide-promoted X-ray energy deposition and the Cu^+^-catalyzed Fenton reaction. Simultaneously, the released thioguanine incorporates into the DNA skeleton, inhibiting the DDR process. As a result, tumor self-defense pathways were disrupted by aaMOF-driven GSH depletion and DDR inhibition, enabling tailored chemoradiotherapy. aaMOF-based radiotherapy exhibits remarkable antitumor efficacy in both cells and a xenograft tumor model. This approach fully leverages the benefits of the all-active MOF components to overcome tumor self-defensive mechanisms and maximise therapeutic outcomes.

## Abstract 2

An all-component-active MOF was designed with copper iodide and thioguanine. Tumor self-defense pathways were disrupted by MOF-driven glutathione depletion and DNA damage repair inhibition under X-ray radiation, enabling tailored chemoradiotherapy.

## Graphical abstract

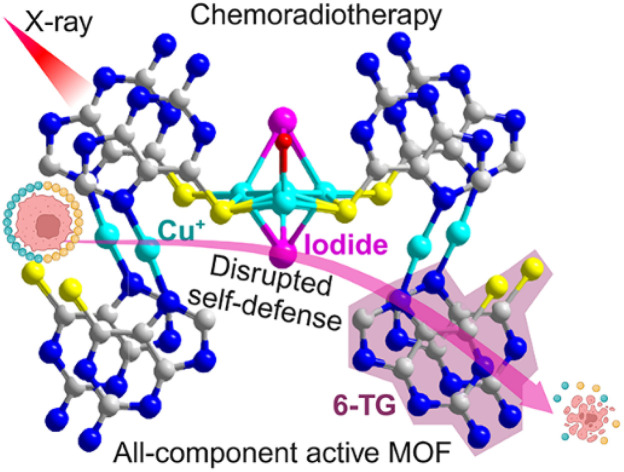

## Introduction

Radiotherapy (RT) is a noninvasive treatment modality that utilizes high-energy radiation (such as γ-rays and X-rays) to directly induce DNA cleavage or indirectly generate reactive oxygen species (ROS) through water radiolysis to destroy DNA.^1–3^ However, abundant glutathione (GSH) and DNA damage repair (DDR) systems in cancer cells can neutralize ROS and restore the structure of DNA, leading to radiation resistance.^4–6^ In addition, excess high-energy radiation could inevitably lead to collateral damage to normal tissues, particularly those exposed to cumulative irradiation.^7–9^ To combat the self-defense pathways of tumors and mitigate serious side effects, various radiosensitizers have been developed to sensitize cancer cells during irradiation.^10^ Heavy-metal-based high-Z materials are promising candidates to act as radiosensitizers due to their relatively large absorption cross sections for high-energy photons.^11–13^ However, using these materials has often been discouraged due to their toxicity.^14,15^ Iodine, a nonmetal, shows promising RT enhancement ability and excellent biocompatibility.^16–18^ Unfortunately, iodine-containing small molecules suffer from rapid clearance and low retention in tumors. To overcome these issues, various vehicles have been employed to enhance the uptake and reduce the efflux of iodine.^19,20^

Except for the use of radiosensitizers, integrating RT with other conventional therapies is another way to fight radiation resistance. Chemoradiotherapy (CRT), which combines radiotherapy with chemotherapy, is one of the most effective treatment regimens for solid tumors.^21–23^ In CRT, ROS generated by high-energy radiation can alleviate desmoplasia, facilitating the permeability of therapeutic agents.^24–27^ Meanwhile, chemotherapeutics such as fluorouracil, irinotecan, and oxaliplatin can inhibit cellular metabolism, weakening the ability of cancer cells to repair damage caused by radiation.^28–31^ However, the systemic administration of most chemotherapeutics often leads to severe toxicity because of their nonspecific distribution in normal tissues.^32^ Nanomaterial-based drug encapsulation systems are extensively applied for targeted delivery, but still limited by low loading capacities and carrier-induced toxicity.^33–35^ Therefore, the careful selection of radiosensitizers, chemotherapeutics, and delivery systems is essential to achieve intelligent CRT that can effectively disrupt tumor self-defensive pathways.^36^

Carrier-free systems have emerged as a promising subdiscipline due to their high drug-loading capacities (even up to 100%), avoidance of carrier-induced toxicity, and simple fabrication processes.^37–39^ Despite these advantages, designing ideal carrier-free radiosensitizers remains challenging due to factors, such as the precise assembly of specific molecules,^40^ the reversibility of weak chemical bonds,^41^ and functionalization difficulties.^42^ Thus, a practical carrier-free radiosensitizer should be rationally designed for CRT involving responsive disassembly and active targeting.^42–44^ Metal–organic frameworks (MOFs), formed through coordination bonds between metal nodes and organic ligands, offer a versatile therapy platform by enabling the multicomponent integration of metal catalysts and photosensitizer.^45,46^ For example, a Hf-MOF consisting of hafnium (Hf) clusters and tetrakis(4-carboxyphenyl) porphyrin has successfully overcome hypoxia-induced radiation resistance.^47^ As a result of their tunable nature, MOFs could deliver radiosensitizers (nodes) and chemotherapeutics (ligands) concurrently in a carrier-free manner, offering a tailored therapeutic solution.^48^

**Scheme 1:**
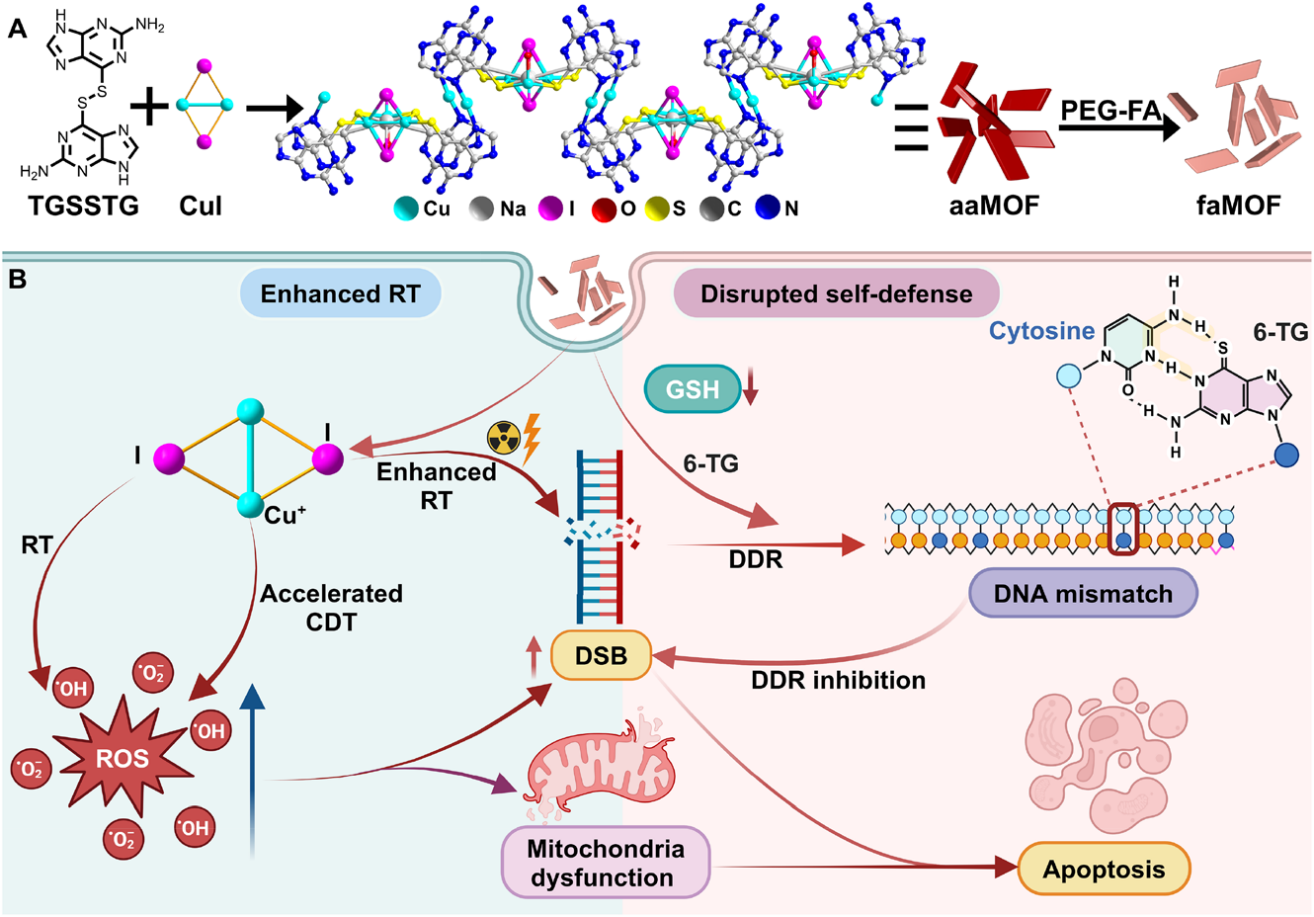
Schematic illustrations of (A) the preparation and functionalization of aaMOF, and (B) the chemoradiotherapy mechanism based on the tumor-responsive degradation of aaMOF acting against the self-defense pathway.

Based on this concept, an all-component-active MOF (aaMOF), with the nucleobase analog thioguanine (6-TG) as the linker and copper iodide as the nodes, is designed to counteract tumor self-defense pathways. Clinically-used 6-TG is selected to construct aaMOF due to its rigid planar structure, abundant coordination sites, and easily functionalized sulfhydryl group.^49–51^ The homodimer 6-TG, *via* a disulfide linkage, (TGSSTG) was synthesized first to serve as the building block for aaMOF. Copper iodide is chosen as the nodes for its efficient chemodynamic therapy (CDT) and X-ray attenuation properties (Scheme 1A). More than just a carrier, aaMOF is precisely tailored through coordination interactions between copper iodide and 6-TG, achieving 100% active-pharmaceutical-ingredient loading and tumor-specific drug release. Upon internalization into cancer cells, polyethylene glycol-folic acid (PEG-FA)-coated aaMOF undergoes endogenous stimuli-responsive disassembly due to the protonation of 6-TG in an acidic environment and competitive coordination with Cu^+^ by overexpressed GSH, releasing 6-TG and copper iodide alongside a reduction in GSH levels (Scheme 1B). Once disassembled, the Cu^+^-catalyzed Fenton reaction and iodide-promoted X-ray energy deposition can generate abundant ROS and DNA double-stranded breaks (DSBs). Meanwhile, as a nucleobase analog, the released 6-TG can be incorporated into the DNA skeleton during the subsequent DDR process, introducing DNA mismatch and inhibiting DDR. As a result, the tumor's defenses can be overcome through aaMOF-inspired GSH depletion and DDR inhibition, augmenting tumor radiation sensitivity and enhancing therapeutic efficacy without appreciable side effects. Therefore, aaMOF paves a new avenue for designing MOF-based all-component-active systems, maximizing therapeutic effects while minimizing side effects.

## Results and discussion

### Synthesis and characterization of aaMOF and faMOF

**Fig. 1:**
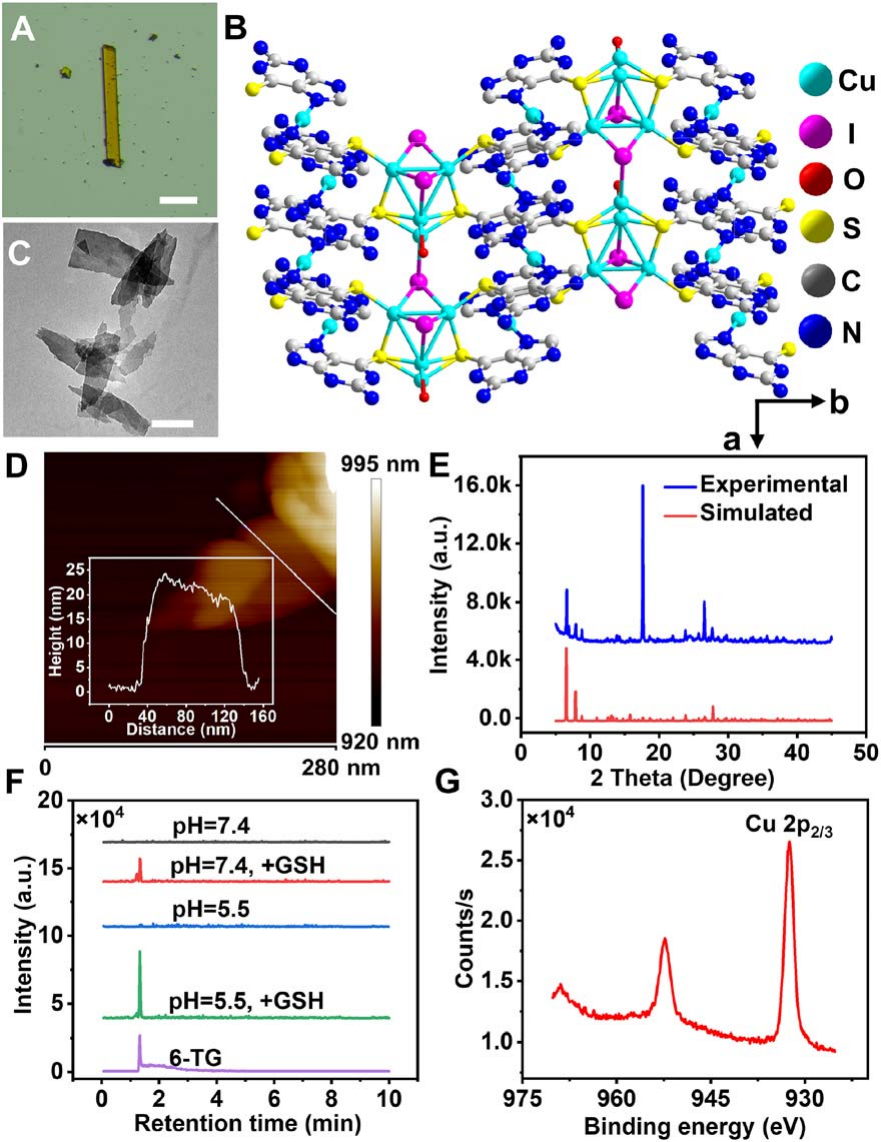
(A) A crystal image of aaMOF; scale bar = 200 μm. (B) The structure of aaMOF along the *c*-axis. (C) A TEM image of aaMOF; scale bar = 100 nm. (D) An AFM image of aaMOF. (E) Experimental and simulated PXRD patterns of aaMOF. (F) LC-MS analysis of standard 6-TG and aaMOF supernatant incubated with and without GSH in PBS at different pHs. (G) The high-resolution Cu 2p XPS spectrum of aaMOF.

aaMOF was synthesized using an *in situ* ligand formation method.^52,53^ At first, 6-TG was transformed into TGSSTG through disulfide linkage,^54^ and the product was successfully isolated with high purity, as verified by ^1^H NMR and liquid chromatograph-mass spectrometry (LC-MS) (Fig S1 and S2†). Using the pre-synthesized TGSSTG ligand and copper iodide as building blocks, aaMOF crystals were finally obtained with a flat parallelogram morphology (Fig. 1A and S3†). The accurate aaMOF structure was determined using single-crystal X-ray diffraction. The monoclinic crystal structure of aaMOF belongs to the *P*2_1_/*m* space group with the chemical formula [(CuI)_2_Cu_0.25_(6-TG)_2_(H_2_O)_0.5_] (CCDC deposition number: 2419561), containing 45.15% 6-TG. In the structure, 6-TG and copper iodide clusters form a zip-like cross connection, creating zigzag chains. As the copper iodide cluster extends, a 2D planar network is constructed, and the MOF is ultimately built *via* hydrogen bonds between these networks (Fig. 1B and S4†). Elemental mapping of aaMOF demonstrated a uniform distribution of N, S, I, and Cu, confirming the homogeneity of the material (Fig. S5†). To enhance cellular uptake, nanoscale aaMOF is synthesized by the addition of five equivalents of copper iodide. Transmission electron microscope (TEM) analysis revealed that nanoscale aaMOF is obtained with a size of *ca.* 200 nm (Fig. 1C). Meanwhile, powder X-ray diffraction (PXRD) and scanning electron microscope analyses confirmed that nanoscale aaMOF presented an isomorphic structure to crystal aaMOF (Fig. S6†). The atomic force microscopy (AFM) profile indicates that the thickness of aaMOF is approximately 20 nm (Fig. 1D). Low nitrogen adsorption/desorption was observed (Fig. S7†), confirming the rigid framework and narrow channels of aaMOF, as supported by crystal analysis. To improve its biocompatibility and targeting abilities, aaMOF was further coated with PEG-FA, resulting in the production of FA-functionalized aaMOF (faMOF). Compared to aaMOF, no obvious morphological changes were observed for faMOF after modification (Fig. S8†). Thermogravimetry analysis of aaMOF displays multistep decomposition, corresponding to the decomposition of 6-TG and the sublimation of iodine at 325 °C and 450 °C, respectively (Fig. S9†).^55^ The ultraviolet spectrum also confirmed the successful assembly of 6-TG and copper iodide, with characteristic absorption peaks from both components (Fig. S10†). Furthermore, the PXRD pattern of aaMOF displays sharp and clear diffraction peaks, indicating the successful preparation of aaMOF with high crystallinity and phase purity (Fig. 1E).

The tumor-specific degradation of aaMOF was verified under simulated, normal and tumor environments. When exposed to GSH and acidic conditions (pH = 5.5), the rapid release of 6-TG was observed, while aaMOF remained stable under normal physiological conditions (Fig. 1F, S11 and S12†). Then, from X-ray photoelectron spectroscopy (XPS) analysis of aaMOF, the Cu 2p_3/2_ peak at 932.45 eV confirmed the presence of Cu^+^ (Fig. 1G, S13 and S14†), which can produce ROS in cancer cells with excess H_2_O_2_.^56^ Upon the introduction of PEG-FA, the surface charge of aaMOF changes from positive to negative, accompanied by the characteristic absorption of folic acid (Fig. S15 and S16†). Different from a single-component example, aaMOF exhibits excellent water dispersibility, and so does faMOF (Fig. S17†). Thus, MOFs with intrinsic stimulus-responsive and active-targeting capabilities were successfully developed.

### ROS production catalyzed by aaMOF

**Fig. 2:**
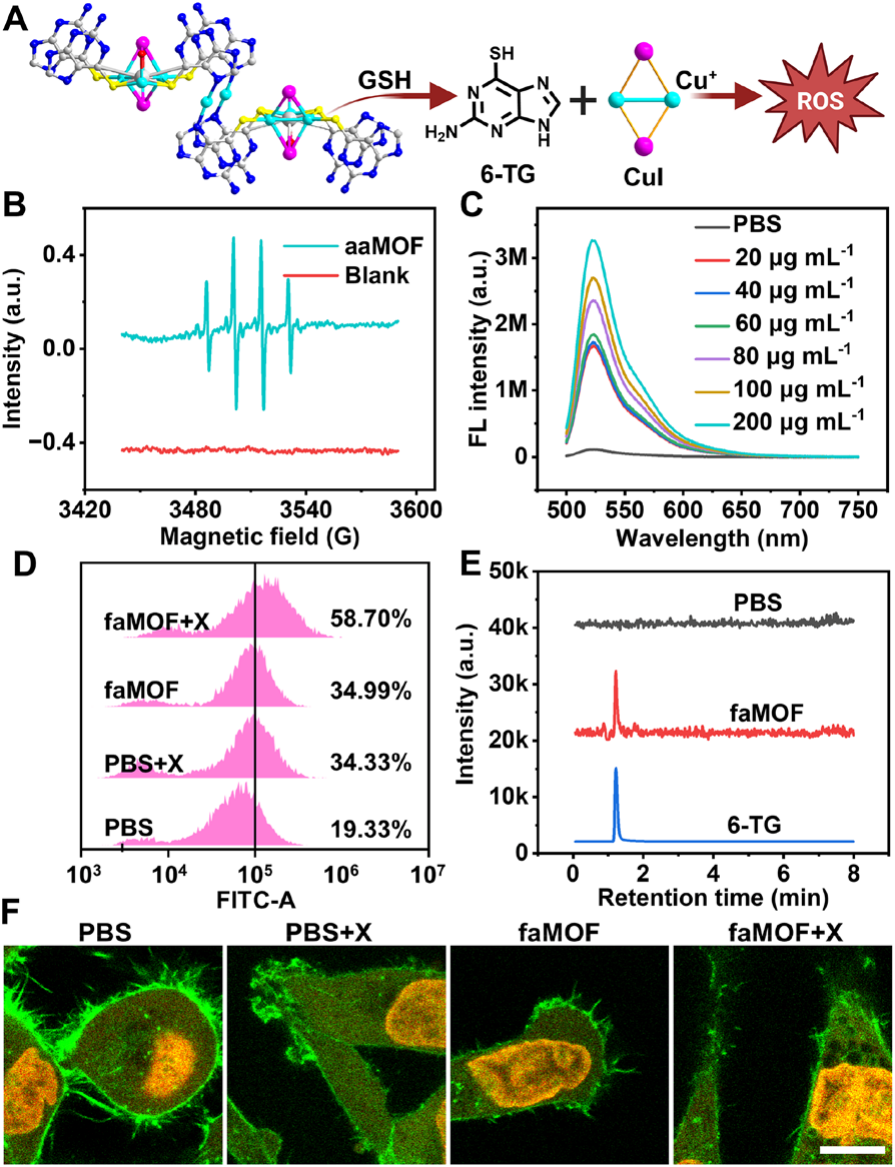
(A) A schematic illustration of ROS production catalysed by Cu^+^ disassembled from aaMOF. (B) The EPR signal of ˙OH using DMPO as a radical scavenger in the presence of aaMOF. (C) Fluorescence spectra of DCFH-DA subjected to X-rays at various concentrations of faMOF. (D) Flow cytometry analysis of cells stained with DCFH-DA after treatment with PBS, PBS + X-ray, faMOF, and faMOF + X-ray. (E) LC-MS analysis of cell supernatants treated with PBS and faMOF. (F) CLSM images of cells treated with PBS, PBS + X-ray, faMOF, and faMOF + X-ray; cells were stained with Hoechst 33342 and rhodamine-labelled phalloidin; scale bar = 10 μm.

CDT has emerged as a promising cancer therapy approach, utilizing Fenton/Fenton-like catalysts to convert intracellular H_2_O_2_ into ROS in tumor microenvironments (Fig. 2A). When aaMOF was incubated with H_2_O_2_, the presence of ˙O_2_^−^ was captured by electron paramagnetic resonance (EPR) using 5,5-dimethyl-1-pyrroline-*N*-oxide (DMPO) as a radical scavenger. The characteristic 1 : 1 : 1 multiplicity of the ˙O_2_^−^-DMPO adduct was immediately observed (Fig. S18†), confirming the generation of ˙O_2_^−^. In addition, a characteristic 1 : 2 : 2 : 1 quartet could be clearly discerned in the EPR spectrum, indicating the formation of ˙OH (Fig. 2B). The accelerated CDT performance of aaMOF was further investigated using the typical ROS substrates 3,3,5,5-tetramethylbenzidine (TMB) and methylene blue (MB). Both TMB and MB rapidly degraded, and CDT activity was significantly accelerated in acidic solution (pH = 5.5) upon adding aaMOF in the presence of H_2_O_2_ (Fig. S19 and S20†). Apart from ROS produced by Cu^+^, iodide-enhanced RT is also an important ROS source. The capacity of faMOF for facilitating X-ray energy deposition was investigated using the ROS indicator 2′,7′-dichlorofluorescin diacetate (DCFH-DA): the total ROS production in the aqueous phase was measured. In the absence of faMOF, only mild fluorescence was observed from DCFH-DA, which significantly increased upon the addition of faMOF (Fig. 2C). Overall, compared to conventional Cu^2+^, Cu^+^ from aaMOF acts as a more efficient Fenton catalyst for CDT under acidic conditions and at elevated H_2_O_2_ levels.^57^

Consistent with *in vitro* measurements, faMOF exhibited exceptional ROS production capabilities in HeLa cells. Intracellular ROS levels were assessed using the fluorescence probe DCFH-DA. Compared to PBS-treated cells, both X-ray and faMOF treatments led to increased green fluorescence (Fig. S21†), indicating the increased production of ROS. The combination of faMOF and X-ray treatment resulted in a threefold increase in ROS-positive cells compared to the PBS group (Fig. 2D), which was attributed to iodide-facilitated X-ray energy deposition. Following the characterization of two components of aaMOF at the cellular level, we next investigated the fate of 6-TG. After treating 6-TG with PBS or faMOF, cell lysates were analyzed by LC-MS for 6-TG. A distinct ion peak identified with standard 6-TG was observed in lysates from faMOF-treated cells, while no such peak was found in PBS-treated cells (Fig. 2E), indicating 6-TG in aaMOF can be responsively released in cancer cells *via* GSH-mediated cellular metabolism. Thus, all components of aaMOF have been proved to be intracellularly active. Moreover, to examine the radiosensitizing effects of faMOF, a morphological survey of HeLa cells was carried out with confocal laser scanning microscopy (CLSM). Altered cell morphologies, such as swelling, cytoplasmic vacuolization, and enlarged nuclei, were observed in X-ray treated cells, and these were further exacerbated in cells treated with both X-rays and faMOF (Fig. 2F and S22†). All the above results support the notion that faMOF is an all-component-active system with promising potential for tailored chemoradiotherapy.

### Sensitizing the RT activity of faMOF

**Fig. 3:**
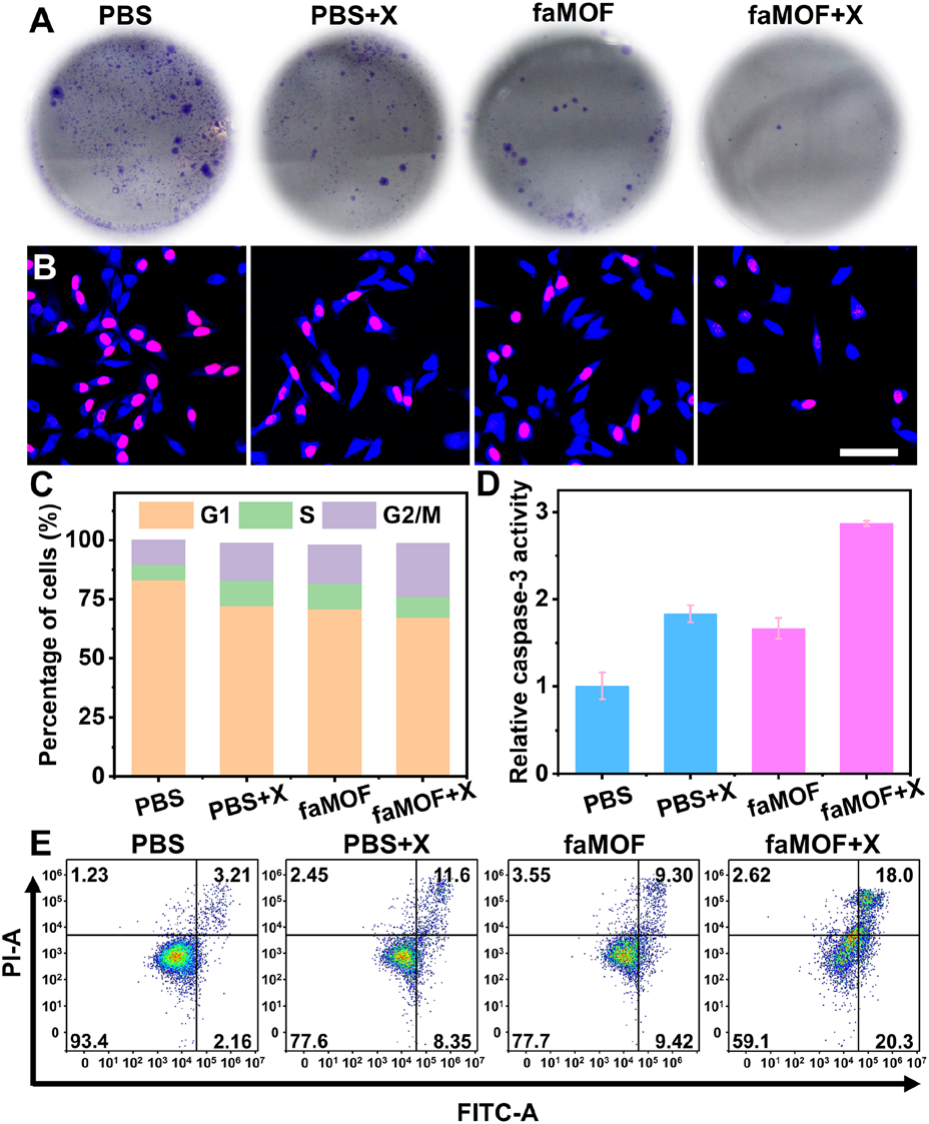
(A) Clonogenic assays of cells stained with crystal violet and (B) CLSM images of cells stained with Hoechst 33342 and Alexa Fluor 594 azide after treatment with PBS, PBS + X-ray, faMOF, and faMOF + X-ray; scale bar = 75 μm. (C) Relative cell ratios in the G2/M, S and G1 phases and (D) relative caspase-3 activities after various treatments. (E) Flow cytometry analysis of the apoptosis of cells treated with PBS, PBS + X-ray, faMOF, and faMOF + X-ray; the percentage of cells is indicated in each quadrant.

To evaluate the therapeutic effects of faMOF, both short-term toxicity (Cell Counting Kit-8, CCK-8) and long-term clonogenic assays were performed. faMOF exhibited excellent tumor cell inhibition efficacy in a dose-dependent manner, with a half-maximal inhibitory concentration (IC_50_) of 0.5 μg mL^−1^ toward HeLa cells when combined with X-ray irradiation (Fig. S23†), exhibiting much higher drug-loading efficiency and therapeutic efficacy than the typical nano-carrier zeolitic imidazolate framework-8 (ZIF-8) (Fig. S24–S28†). The resulting low dosage signifies effectiveness and protection against adverse reactions involving aaMOF. In clonogenic assays, faMOF effectively reduced colony numbers and sizes after the cells were exposed to X-rays (Fig. 3A and S29†), outperforming X-ray treatment alone (Fig. S30†). The reduced cell-cloning density and survival fraction indicated that faMOF is an efficient radiosensitizer for RT. To validate the potential therapeutic efficacy of faMOF, the cell proliferation ability was evaluated using 5-ethynyl-2′-deoxyuridine (EdU), which can insert itself into DNA molecules when cells proliferate. Benefitting from iodide-enhanced X-ray energy deposition, a significant reduction in EdU fluorescence was observed in the faMOF + X-ray group (Fig. 3B). Therefore, faMOF afforded the notable inhibition of DNA duplication owing to ROS-produced DNA damage and 6-TG-introduced DNA mismatch.^58^

Given that DNA duplication is a key event in the cell cycle for producing two daughter cells, we analyzed the cell cycle distribution of faMOF-treated cells. X-ray treatment alone induced a moderate the cell cycle arrest (Fig. 3C), resulting in a 1.5-fold increase in the percentage of G2/M-stage cells. When faMOF was added, the cell cycle was remarkably arrested in the G2/M phase, two-fold more than in the PBS group, indicating the inhibition of mitosis. Usually, a stagnant cell cycle activates the apoptosis pathway,^59^ and, as the key modulator of apoptosis, the expression of caspase-3 was investigated. As expected, a three-fold elevation in caspase-3 activity in cancer cells was observed after treatment with faMOF and X-ray irradiation (Fig. 3D). In contrast, the caspase-3 activity remained at a basal level in the control group. Additionally, cell apoptosis was quantified through flow cytometry (Fig. 3E). The PBS-treated group presented negligible proportions of apoptotic cells, while the proportion of apoptotic cells (about 20%) was significantly higher upon treatment with X-rays or faMOF. Remarkably, a two-fold increase in apoptotic cells was seen in the faMOF + X-ray group. According to the above results, faMOF showed outstanding therapeutic efficiency and excellent properties for sensitizing RT.

### Therapeutic mechanism of all-component-active faMOF

**Fig. 4:**
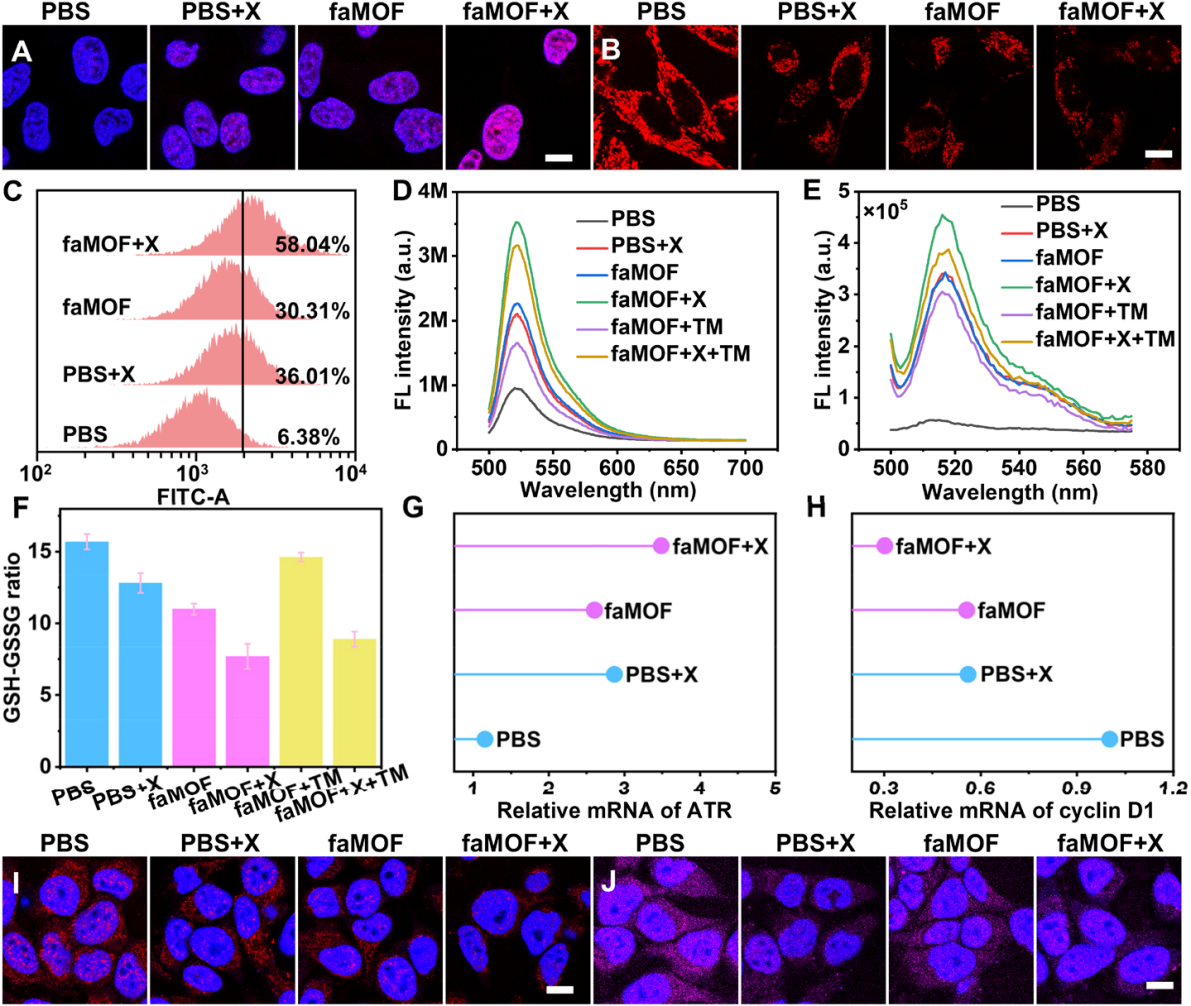
Representative CLSM images of (A) cell immunofluorescence against γH2AX, and (B) cells stained with Hoechst 33342 and TMRE after various treatments; scale bar = 10 μm. (C) Flow cytometry analysis of cells stained with BODIPY-C11 after treatment with PBS, PBS + X-ray, faMOF, and faMOF + X-ray. Fluorescence spectra of cell lysates stained with (D) DCFH-DA and (E) BODIPY-C11 after various treatments. (F) GSH-GSSG ratios and relative (G) ATR and (H) cyclin D1 mRNA expression levels in cells subjected to various treatments. CLSM images of (I) HIF and (J) survivin immunofluorescence analysis; scale bars = 10 μm.

To further explore the therapeutic mechanism of faMOF, the key hallmarks of oxidative stress and apoptosis pathways were surveyed. To verify DNA damage induced by ROS and enhanced RT, we studied the formation of phosphorylated histone H2AX (γH2AX), an early cellular response of DSBs.^60^ As indicated by the bright fluorescence, a substantial increase in the γH2AX level was observed in the faMOF + X-ray group (Fig. 4A), suggesting that combined treatment can induce abundant DSBs. This hypothesis was further confirmed by the observation of increased expression of poly(ADP-ribose) polymerase (PARP), which is activated by DSB to repair DNA damage, following faMOF + X-ray treatment (Fig. S31†).^61^ Except for inducing DNA damage, ROS generated by Cu^+^ and X-ray irradiation can also destroy cancer cells by oxidative stress. Accumulated ROS is known to interact with lipids, inducing lipid damage through lipid peroxide (LPO), which disrupts cell membrane integrity.^62^ The number of LPO-positive cells in the faMOF + X-ray group was found to be elevated from 6.38% to 58.04% compared to the PBS group (Fig. 4C and S32†), which means faMOF-enhanced CRT can effectively disrupt the cell membrane integrity. Apart from triggering LPO, ROS also eliminated cancer cells *via* the mitochondria apoptosis pathway.^63^ Moreover, the mitochondrial membrane potential (MMP) was measured using the fluorescence probe tetramethylrhodamine ethyl ester perchlorate (TMRE). The decrease in the red fluorescence intensity of faMOF- and X-ray-treated cells reflects the elimination of the MMP (Fig. 4B). Besides, the mitochondria of cells in the faMOF + X-ray group showed a smaller size, shrunken morphology, and reduced number, indicating ROS-induced mitochondrial dysfunction in cancer cells.

To confirm the role of iodide in promoting ROS production, the Cu^+^-catalyzed Fenton reaction was inhibited using the copper-specific chelator tetrathiomolybdate (TM). Then the total ROS and LPO content levels in cells were assessed by corresponding fluorescence probes. Compared to the faMOF + X-ray group, the total ROS and LPO levels in the faMOF + X-ray + TM group showed a certain degree of reduction but were much higher than those of the faMOF + TM group (Fig. 4D and E), confirming that iodide facilitates X-ray energy deposition and ROS generation. Additionally, the GSH-GSSG ratio was measured to evaluate the cellular oxidative stress status. As an important ROS scavenger, GSH reacts with ROS to produce oxidized glutathione (GSSG). A reduction in the GSH-GSSG ratio reflects reduced antioxidant capacity.^64^ Cells treated with faMOF displayed a GSH-GSSG ratio decrease of approximately one-third due to competitive coordination and the ROS production capacity of Cu^+^ (Fig. 4F). The lowest GSH-GSSG ratio (less than half of the PBS group) was observed in the faMOF + X-ray group, which can be attributed to iodide-facilitated X-ray energy deposition and ROS generation.^65^ An evaluation of relative GSH levels showed similar results (Fig. S33†), further confirming the competitive coordination effects of Cu^+^ and iodide-amplified ROS production.

Oxidative stress and DNA damage should activate proteins involved in DNA repair, cell proliferation and apoptosis.^66^ Cells transmit DNA damage signals to DNA repair kinases, such as ataxia telangiectasia and Rad3-related protein (ATR), blocking cell cycle progression and initiating the repair of damaged DNA.^67^ Gene expression levels were quantified by quantitative real-time polymerase chain reaction and western blot analysis. ATR mRNA and protein levels increased (approximately 3.5-fold for mRNA) in cells treated with X-ray irradiation, indicating the activation of the ATR-mediated DNA repair pathway in response to faMOF-induced DNA damage (Fig. 4G and S34†). Moreover, as a marker of cell proliferation, cyclin D1 mRNA levels were reduced (by about 70% for mRNA) in the faMOF + X-ray group (Fig. 4H),^68^ and so was cyclin-dependent kinase 2 (Fig. S35†), which drives the cell cycle.^69^ Overall, the delayed cell cycle and inhibited cell proliferation were induced by both DNA damage resulting from ROS and DDR inhibition caused by 6-TG.

As mentioned above, faMOF can kill cancer cells not only by producing ROS and sensitizing radiotherapy but also by depleting GSH and inhibiting DDR, overcoming a tumor's defenses. As a hallmark of radiation resistance, hypoxia inducible factors (HIF) significantly contribute to RT resistance by promoting the expression of genes involved in cell survival, DNA repair, and anti-apoptotic pathways.^70^ Therefore, we assessed cellular HIF-1α levels by immunofluorescence staining. Benefiting from 6-TG-caused RNA transcription and endogenous HIF target gene inhibition,^71,72^ decreased HIF-1α levels were observed in faMOF + X-ray treated cells (Fig. 4I), implying a hurdle in evading apoptosis. Regulated by HIF, survivin suppresses caspase activation upon the X-ray irradiation of cancer cells.^73^ The results of survivin immunofluorescence analysis demonstrated a reduction in survivin in cells treated with faMOF + X-ray as compared with the negative control group (Fig. 4J), indicating that faMOF-treated cells are highly sensitive to radiation damage. Therefore, the therapeutic mechanism of faMOF involves integrating the activities of all the components of faMOF. That is, it benefits from the Cu^+^-catalyzed Fenton reaction and iodide-promoted RT, leading to abundant ROS generation and irresistible DSB in cancer cells. In addition, 6-TG released from faMOF can block DDR, overcoming the radiation resistance of cancer cells, while GSH was reduced due to competitive coordination with Cu^+^ and the generation of intracellular ROS, thereby disrupting the tumor's self-defense pathway.

### Therapeutic efficacy against self-defensive tumors

**Fig. 5:**
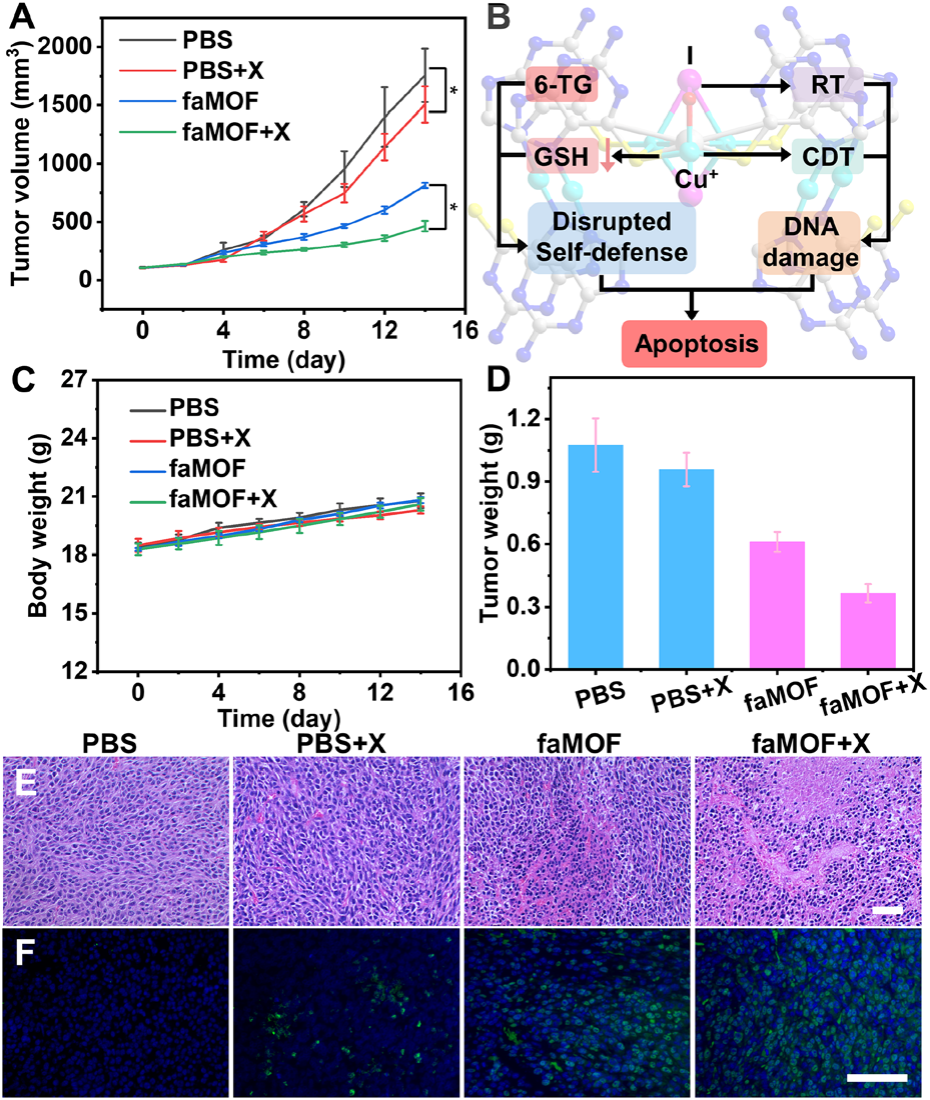
(A) The tumor growth profiles of each group during therapeutic progress (*n* = 5). (B) A schematic illustration of the therapeutic pathway of faMOF. (C) Mice weight curves (*n* = 5) during therapy. (D) The weights of tumors extracted from mice 14 days after the beginning of treatment. (E) Representative H&E staining and (F) immunofluorescence images of γH2AX in tumor tissues after various treatments; scale bars = 50 μm.

Based on the excellent therapeutic efficacy and RT-enhancing effect of faMOF in cells, we further evaluated the therapeutic efficacy of faMOF*in vivo*. Tumor-bearing mice were established using HeLa cells. These mice were randomly divided into four groups when the tumor size reached ∼100 mm^3^. Tumor volumes in the PBS treatment group increased rapidly (Fig. 5A), while tumor growth was inhibited to varying degrees in the PBS + X-ray, faMOF, and faMOF + X-ray groups. Remarkably, significant tumor weight loss was observed in the faMOF + X-ray group, where the tumor inhibition rate was 66%,attributed to faMOF-promoted CRT enhancement and the disruption of self-defense mechanisms (Fig. 5B). Mice weights remained stable in all groups during the whole therapeutic process (Fig. 5C), and no apparent pathological abnormalities were observed on the major organs (Fig. S36†), suggesting the favorable biocompatibility of faMOF. Overall, a synergistic effect was obtained by aaMOF-based CRT (Fig. 5D and S37†). Based on the hematoxylin–eosin (H&E) analysis of tumor sections, severe nucleus dissociation and necrosis of cancer cells were observed (Fig. 5E), implying the critical therapeutic role of faMOF toward tumors. Additionally, immunofluorescence staining against PARP and γH2AX was performed; bright fluorescence is distributed in tumor sections upon faMOF and X-ray treatments, leading to the prominent upregulation of PARP and γH2AX (Fig. 5F and S38†). Consequently, conspicuous therapeutic effects based on all-component-active MOFs were achieved by the simultaneous enhancement of CRT and the disruption of tumor defense mechanisms *in vivo*.

## Conclusions

In summary, an all-component-active MOF is successfully designed with maximum therapeutic effects and minor side effects for CRT against tumor self-defenses. Using copper iodide as the nodes and clinically-used 6-TG as the ligand, aaMOF was synthesized with 100% active-pharmaceutical-ingredient loading and tumor-specific drug release, thereby avoiding carrier-induced toxicity. Due to the protonation of 6-TG under acidic conditions and the competitive coordination with Cu^+^ of intracellular GSH, aaMOF undergoes tumor-specific degradation, releasing all copper iodide and 6-TG components. The Cu^+^-catalyzed Fenton reaction cooperates with iodide-enhanced RT, generating abundant ROS and DSBs. Meanwhile, the released 6-TG can block DDR, and there is a reduction in GSH both due to competitive coordination with Cu^+^ and the consumption of intracellular ROS, breaking the self-defense pathway of cancer cells. Consequently, remarkable therapeutic efficacy was realized by synchronizing enhanced CRT and disrupted self-defenses in a xenograft tumor model. Our work provides new insight for designing all-component-active MOF platforms for maximizing therapeutic effects against tumor self-defenses.

## Ethics statement

All animal procedures were performed in accordance with the Guidelines for the Care and Use of Laboratory Animals of Nanjing University and approved by the Animal Ethics Committee of Model Animal Research Center (No. IACUC-2502007).

## Author contributions

X. Xu and J. P. Lei proposed the idea and designed the experiments. X. Xu, Z. Yang and J. P. Lei wrote the manuscript. X. Xu carried out the synthesis of nanoagents and cell experiments. S. S. Bao helped in crystal analysis. Z. Y. Xu and T. R. Liu helped in scanning/transmission electron microscope measurements. J. P. Lei and L. N. Wu supervised and coordinated all investigators for this project. All authors discussed the results and commented on the manuscript.

## Conflicts of interest

The authors declare no competing interests.

## Supplementary Material

SC-016-D5SC02482J-s001

SC-016-D5SC02482J-s002

## Data Availability

The data supporting this article have been included as part of the ESI.†
