# Supplementary material for: All-component-active metal–organic frameworks for tailored chemoradiotherapy of self-defensive tumors
Source: Chem Sci. 2025 Jun 26;16(31):14295–303. doi: 10.1039/d5sc02482j (PMC12242832; doi:10.1039/d5sc02482j)
Supplement: SC-016-D5SC02482J-s001 [file SC-016-D5SC02482J-s001.pdf]

Supplementary Information (SI) for Chemical Science.
This journal is © The Royal Society of Chemistry 2025

Supplementary Information

All-component active metal-organic frameworks for tailored chemoradiotherapy of self-defensive tumor

Xiang Xu,[a] Zhou Yang,[a] Songsong Bao,[a] Zhiyuan Xu,[a] Tianrui Liu,[a] Lina Wu*[b] and Jianping Lei*[a]

[a]State Key Laboratory of Analytical Chemistry for Life Science, State Key Laboratory of Coordination Chemistry, School of Chemistry and Chemical Engineering, Nanjing University, Nanjing 210023, China

[b]State Key Laboratory of Microbial Technology, School of Food Science and Pharmaceutical Engineering, Nanjing Normal University, Nanjing 210023, China

* Corresponding author. E-mail: wuln@njnu.edu.cn (L Wu), jpl@nju.edu.cn (J Lei)

Experimental Section

Materials and reagents. Thioguanine (6-TG), 5,5-dimethyl-1-pyrroline N-oxide (DMPO), and formic acid were purchased from Sigma-Aldrich (Merck, USA). Ethylene glycol, N-hydroxy succinimide-polyethylene glycol-folic acid (NHS-PEG-FA), and copper iodide were obtained from Sinopharm Chemical Reagent Co., Ltd. and used directly without further purification. The cell culture media (Dulbecco's Modified Eagle Medium, DMEM), trypsin-EDTA (ethylenediaminetetraacetic acid), and penicillin/streptomycin were from SenBeiJia Biological Technology Co., Ltd. (China). Dimethyl formamide, sodium bicarbonate, and iodine were purchased from Bidepharm. Cell Counting Kit-8 (CCK-8), EdU (5-Ethynyl-2'-deoxyuridine) cell proliferation kit, rabbit polyclonal antibody (anti-PARP, anti-H2AX, anti-HIF, anti-survivin), and the Alexa Fluor 647-conjugated secondary antibody (anti-rabbit secondary antibody) were obtained from Beyotime Technology Co., Ltd. (China). Tetramethylrhodamine ethyl ester (TMRE), 2',7'-dichlorodihydrofluorescein diacetate (DCFH-DA), and BODIPY-C11 were purchased from InvivoChem. Primers were purchased from Sangon Biotech (Shanghai) Co., Ltd. RNA reverse transcription kit, and qPCR kit were purchased from Beijing Tsingke Biotech Co., Ltd. All aqueous solutions were prepared using ultrapure water ($\geq$ 18 MΩ·cm, Milli-Q, Millipore).

Characterizations. The transmission electron microscopic (TEM) images were recorded on a JEM-2100 high resolution transmission electron microscope (JEOL Ltd., Japan). The zeta potential was measured on Nano-Z Zetasizer (Malvern Panalytical, UK). Powder X-ray diffraction (PXRD) data was obtained by a X'TRA diffractometer (ARL, Switzerland). The ultraviolet absorption spectra were obtained with a UV-3600 UV-Vis-NIR spectrophotometer (Shimadzu Co., Japan). CCK-8 assays were carried out on a Synergy hybrid 1 multimode microplate reader (BioTek). The cell images were gained on a TCS SP8 laser scanning confocal microscope (Leica, Germany). Flow cytometric analysis was performed on a Coulter FC-500 flow cytometer (Beckman-Coulter). High resolution mass spectra were run on an Agilent 6530 TOF LC/MS mass spectrometer. ^{1}H liquid-state NMR spectra were recorded on a Bruker AVANCE spectrometer (400 MHz) using dimethylsulfoxide-d_6 (DMSO-d_6) as the solvent and tetramethylsilane (TMS) as an internal reference. ATR-FTIR spectra were collected with a Nicolet iS50 FTIR spectrometer (Thermo). XPS was performed with a Thermo Scientific Nexsa using monochromatic Al K_α radiation

(1,486.68 eV) and a low-energy food gun as a neutralizer. All the binding energies were calibrated using the C 1s peak (BE = 284.8 eV) as standard. Gas sorption measurements were conducted using a Micromeritics ASAP 2460 system, and the powder samples were pretreated by degassing at 100 °C under vacuum for 4-6 h. Thermogravimetric analysis (TGA) data were obtained on a TGA 4000 thermal analysis system at a heating rate of 5 $°C \cdot min^{-1}$ under air atmosphere. Atomic force microscope (AFM) images were collected by Dimension Icon Bruker.

In this radiotherapy session, a Varian Clinac 23EX linear accelerator (LINAC) was used to deliver 6 MV high-energy X-rays under an intensity-modulated radiotherapy (IMRT) protocol. The radiation field size was set to 33.0 cm × 14.4 cm, with a total of 400 monitor units (MU) administered at a dose rate of 400 MU/min. The total treatment time was 1.47 min, and no accessories were used. The estimated delivered dose was approximately 4 Gy.

Synthesis of TGSSTG. Homodimer of 6-TG linked by disulfide bond (TGSSTG) was synthesized according to previous report with little modifications.[S1] Briefly, 1.4 g thioguanine was dispersed in 300 mL of saturated sodium bicarbonate solution under heating, then the mixture was allowed cooled to room temperature. After that, 2.1 g I_2 was dissolved in 8 mL of DMF and dropped into abovementioned mixture dropwise. The system was further stirred 4 h and sediment was collected by centrifugation. The sediment was washed with water several times and dried under vacuum.

All-component active metal organic framework (aaMOF). Briefly, 8.0 mg TGSSTG and 4.75 mg copper iodide were dispersed in 1.5 mL ethylene glycol, then 1.0 mL distilled water and 0.15 mL formic acid were added. The mixture was placed in an oven kept at 90 °C for 72 h to yield an orange crystal. The products were collected and washed with water for several times to remove impurities as much as possible. Finally, the product was dried at 50 °C under vacuum. For obtaining the nanoscale **aaMOF**, five equivalent copper iodide was added for biological experiments.

PEG-FA functionalized aaMOF (faMOF). 15 mg **aaMOF** was dispersed in 50 mL of distilled water, then 15 mg NHS-PEG-FA was dissolved in 10 mL water and added dropwise. After 12 h stirring, the product was harvested by centrifugation and washed several times with water to remove residual PEG-FA. Finally, the product was dried at 50 °C under vacuum.

Synthesis of ZIF-8 and 6-TG@ZIF-8. Briefly, for synthesis of ZIF-8, 0.117 g $Zn(NO_3)_2 \cdot 6H_2O$ was dissolved in 0.8 mL H_2O. Then, a solution of 2.27 g 2-methylimidazole in 8 mL H_2O was prepared. The solution was stirred and 1.2 mL DMSO was added. Zinc nitrate solution was then added to the mixture, followed with 5 min stirring. Then the product was collected by centrifugation and washed with water for several times. Finally, the product was dried at 50 °C under vacuum. 6-TG@ZIF-8 was synthesized in the similar way. In the typical procedure, 10 mg 6-TG was dissolved in 1.2 mL DMSO prior to its addition in the linker solution.

GSH-responsive drug release. faMOF with a concentration of 40 μg mL^{-1} was dispersed in PBS containing 10 mM GSH at pH=7.4 or pH=5.5, and then the resulting mixture was stirred for 4 h, followed by centrifugation. Liquid supernatant was submitted to LC-MS analysis while pure thioguanine was analyzed as control.

aaMOF-catalyzed ROS production. ROS production catalyzed by **aaMOF** was verified by methylene blue and 3,3',5,5'-tetramethylbenzidine (TMB) degradation method. Briefly, the reaction was carried in a 200 μL system containing 5 μg mL^{-1} **aaMOF**, 8 mM H_2O_2, and 30 μg mL^{-1} methylene blue in HEPES at various pHs. After incubated 1 h, the degradation of methylene blue was measured by UV-Vis-NIR spectrophotometer. As for TMB degradation, abovementioned mixture was continued to use except for adopting 10 μg mL^{-1} TMB, and the mixture was immediately measured by UV-Vis-NIR spectrophotometer without further incubation.

Electron paramagnetic resonance. In a typical measurement, 1.0 mg mL^{-1} **aaMOF**, 0.8 M H_2O_2 and 100 μg mL^{-1} DMPO were prepared in aqueous solutions. Then a mixture with the volume ratios of 5:1:10 was confected. The abovementioned mixture was added to 100 μL of MeOH for O_2^{-}• and 100 μL of H_2O for •OH measurement, respectively.

Cell culture. Human cervical cancer cell line (HeLa) was provided by the Institute of Basic Medical Sciences, Chinese Academy of Medical Sciences. Cells were cultured in DMEM supplemented with 10% (v/v) fetal bovine serum (FBS), penicillin (100 U mL^{-1}) and streptomycin (100 μg mL^{-1}) at 37°C in a humidified incubator under 5% CO_2. The cells were routinely harvested by the use of a trypsin-EDTA

solute on (0.25%) until the confluence was reached. In a typical experiment, 1.0 μg mL^{-1} **faMOF** was adopted to treat cells.

Cell morphology. Cells were seeded in 35-mm confocal dish at the density of 1×10^4 cells per well. After adherent, the cells were cultured with PBS or **faMOF** for 8 h. Cells were exposure to 4 Gy X-ray and further cultured for 20 h. The cells were incubated with 100 nM phalloidin–Rhodamine and Hoechst 33342 for 1 h. The CLSM images were collected at 420-500 nm under the 405 nm excitation for DAPI channel, and 570-585 nm under the 543 nm excitation for Rhodamine channel.

Cellular release of 6-TG. Cells were seeded in 6-well plates at a density of 1×10^5 cells per well for 24 h. Then the cells were incubated with PBS or 1.0 μg mL^{-1} **faMOF** for 8 h. Cells were washed twice with PBS and detached by trypsinization. Then cells were resuspended with 100 μL PBS and fractured by ultrasound. The supernatant of cell lysis was submitted to LC-MS analysis with free 6-TG as standard sample.

In vitro cytotoxicity assay. *In vitro* cytotoxicity was assessed by the standard CCK-8 assay. Firstly, cells were seeded in 96 well-plates at a density of 5×10^3 cells per well for 24 h. Then the cells were incubated with PBS or different concentration of **faMOF**. After incubated for 8 h, cells were exposure to 4 Gy X-ray and further incubated for 20 h. Then the medium was removed, and 100 μL of fresh medium with 10 μL of CCK-8 solution were added and incubated for another 1 h. The absorbance at 450 nm was measured to calculate the cell viability by a microplate reader. The statistical evaluation of data was performed using a two-tailed unpaired Student's t-test. Each data point is represented as mean ± standard deviation (SD) of independent experiments (n = 6, n indicates the number of wells in a plate for each experimental condition).

Clonogenic assay. Cells were seeded in 6-well plates at a density of 5×10^3 cells per well for 24 h. Then the cells were incubated with PBS or **faMOF** for 8 h. Cells were exposure to 4 Gy X-ray and further incubated for 20 h. After cells were subsequently propagated for 7 days in **faMOF**-free medium, which was changed every 3 days. Then cells were stained with crystal violet. Briefly, cells were fixed with 10% formalin for 30 min at room temperature, followed with staining by 0.1% crystal violet in 70% ethanol (600 μL per well) for 30 min. Next, cells were washed with deionized water three times for 5 min each to

remove unbounded crystal violet. Finally, crystal violet dye was extracted with 300 μL of 10% acetic acid, and 80 μL was transferred into a 96-well format in triplicate for quantitation.

Cell proliferation assay. Cells were seeded in 35-mm confocal dish at the density of 1 × 10^4 cells per well. After adherent, the cells were cultured with PBS or **faMOF** for 8 h. Cells were exposure to 4 Gy X-ray and further incubated for 20 h. Then cells were washed with PBS several times and cultured with fresh medium. EdU (10 μM) was added to the culture medium, and cells were incubated at 37°C for 2 h. Cells were washed with PBS and fixed with 3.7% formaldehyde at room temperature for 20 min. Following fixation, cells were washed with washing buffer three times (5 min each time). After incubated with permeabilization buffer for 30 min, cells were washed three times (5 min each time) with washing buffer, then 0.5 mL Click-iT reaction mixture (1× Click-iT reaction buffer, $CuSO_4$, Alexa Fluor 594 Azide, and reaction buffer additive) (Beyotime) was added to each well and cells were incubated at room temperature for 30 min in the dark. Cells were washed three times (5 min each time) with washing buffer and incubated with DAPI (0.5 μg mL^{-1} in PBS), then cells were washed with washing buffer and soaked in 3.7% formaldehyde. The CLSM images were collected at 420-500 nm under the 405 nm excitation for DAPI channel, and 610-620 nm under the 590 nm excitation for Alexa Fluor 594 Azide channel.

Relative caspase-3 activity. The HeLa cells were seeded in the 6-well plates at the density of 1 × 10^5 cells per well. After adherent, the cells were cultured with PBS or **faMOF** for 8 h. Cells were exposure to 4 Gy X-ray and further incubated for 20 h, then cells were harvested. After lysed with 200 μL lysis buffer, Ac-DEVD-AMC was added into cell lysis at a final concentration of 20 μM. The lysis was incubated in 37°C for 1 h and fluorescence intensity was measured at λ_{exc} 360 nm and λ_{em} 460 nm.

Flow cytometry analysis of apoptosis. Cells were seeded 6-well plates at a density of 1 ×10^5 cells per well for 24 h. After adherent, cells were cultured with PBS or **faMOF** for 8 h. Cells were exposure to 4 Gy X-ray and further incubated for 20 h. The culture medium was centrifuged to harvest apoptotic cells. Then, cells on plates were washed twice and detached by trypsinization. After cells were washed twice with PBS and stained with FITC-Annexin V and PI for 10 min, the flow cytometry was carried out.

ROS, LPO, MMP visualization. HeLa cells were seeded in 35-mm confocal dish at the density of 1 × 10^4 cells per well. After adherent, cells were cultured with PBS or **faMOF** for 8 h. Cells were exposure

to 4 Gy X-ray and further incubated for 20 h. For ROS, DCFH-DA was added at a final concentration of 20 μM. Cells were further incubated for 1 h, and washed with PBS several times. The CLSM images were collected at 525 nm under the 488 nm excitation. For LPO (lipid peroxide), C11-BODIPY was added at a final concentration of 5 μM. Cells were further incubated for 1 h, and washed with PBS several times. The CLSM images were collected at 510 nm under the 500 nm excitation. For mitochondrial membrane potential (MMP), tetramethylrhodamine ethyl ester perchlorate (TMRE) was added at a final concentration of 50 nM. Cells were further incubated for 1 h, and washed with PBS several times. The CLSM images were collected at 575 nm under the 549 nm excitation.

Flow cytometry analysis of ROS and LPO. Cells were seeded 6-well plates at a density of 1×10^5 cells per well for 24 h. After adherent, cells were cultured with PBS or **faMOF** for 8 h. Cells were exposure to 4 Gy X-ray and further incubated for 20 h. After the culture medium was removed, cells were washed by PBS and incubated with 20 μM DCFH-DA or 5 μM BODIPY-C11, and then detached by trypsinization. Finally, cells were washed twice with PBS and tested with flow cytometry.

GSH determination. The HeLa cells were seeded in the 6-well plates at the density of 1×10^5 cells per well. After adherent, the cells were cultured with PBS or **faMOF** for 8 h. Cells were exposure to 4 Gy X-ray and further incubated for 20 h, then cells were harvested. After lysed by repeated cycles of freezing and thawing, the treated cells were centrifuged under 10000 g and 4 °C for 10 min. Then, GSH level in the supernatant was measured by a GSH detection kit.

Immunofluorescence. Cells were seeded in 35-mm confocal dish at the density of 1×10^4 cells per well. After adherent, the cells were cultured with PBS or **faMOF** for 8 h. Cells were exposure to 4 Gy X-ray and further incubated for 20 h. Then cells were washed with PBS several times and fixed with 3.7% formaldehyde at room temperature for 20 min. Following fixation, cells were washed with washing buffer three times (5 min each time). After incubated with permeabilization buffer for 30 min, cells were washed three times (5 min each time) with washing buffer, followed by incubated with blocking buffer for 1 h. Cells were washed three times (5 min each time) with washing buffer, then primary antibody diluted at a ratio of 1:200 (PARP, H2AX) (Beyotime) was added to each well and cells were incubated at 4 °C for overnight. After washed three times (5 min each time) with washing buffer, cells were incubated with

secondary antibody conjugated with Alexa Fluor 647 for 2 h. Then cells were washed three times (5 min each time) with washing buffer and incubated with DAPI (0.5 μg mL^{-1} in PBS). After washed with washing buffer, cells were soaked in 3.7% formaldehyde. The CLSM images were collected at 420-500 nm under the 405 nm excitation for DAPI channel, and 660-675 nm under the 651 nm excitation for Alexa Fluor 647 channel.

Chelation of Cu^{+}. Cells were seeded 6-well plates at a density of 1 $\times 10^5$ cells per well for 24 h. After adherent, cells were cultured with PBS, **faMOF** and **faMOF**+30 μM tetrathiomolybdate for 8 h. Cells were exposure to 4 Gy X-ray and further incubated for 20 h. Then cells were washed twice with PBS and incubated with DCFA-DA or LPO, followed with detached by trypsinization. These cells were resuspended with 100 μL lysis buffer for further survey.

Flow cytometry analysis of cell cycle. Cells were seeded 6-well plates at a density of 1 $\times 10^5$ cells per well for 24 h. After adherent, cells were cultured with PBS or **faMOF** for 8 h. Cells were exposure to 4 Gy X-ray and further incubated for 20 h. Then, cells were washed twice with PBS and detached by trypsinization. Cells were harvested and washed twice with cold PBS. After soaked in cold 70% ethanol (-20°C) for 4 h, cells were washed twice with PBS followed by incubated with RNase A and PI for 1 h. At last, the cells were tested with flow cytometry and cell cycle was determined by the contents of DNA.

Quantification of ATR, CDK2 and Cyclin D1. Cells were seeded 6-well plates at a density of 1 $\times 10^5$ cells per well for 24 h. After adherent, cells were cultured with PBS or **faMOF** for 8 h. Cells were exposure to 4 Gy X-ray and further incubated for 20 h. Then, cells were washed twice and detached by trypsinization. Total RNA was extracted using a RNA extraction kit, and reversed to cDNA. The cDNA was used for qPCR, GAPDH was set as the reference gene. Primers were list as follows: GGAGATTTCCTGAGCATGTTCGG (ATR-F); GGCTTCTTTACTCCAGACCAATC (ATR-R); CCAGGAGTTACTTCTATGCC (CDK2-F); ATAGTGCAGCATTTGCGA (CDK2-R); GCGGAGGAGAACAAACAGAT (Cyclin D1-F); TGAACTTCACATCTGTGGCA (Cyclin D1-R); GCCTCAAGATCATCAGCAAT (GAPDH-F); TTCAGCTCAGGGATGACCTT (GAPDH-R).

Therapeutic treatment in vivo. All animal assays obeyed the institutional animal use and care regulations approved by the Model Animal Research Center of Nanjing University (MARC). To establish

the tumor bearing xenograft mouse model, female BALB/c mice (6-8 weeks-old) were chosen to be inoculated with HeLa cells (1.0 × 10^6) subcutaneously into the right flank position. Tumor sizes were measured using a digital caliper. The tumor volumes were calculated using formula $V = 0.5 \times A \times B^2$ (A refers to the tumor length and B refers to the tumor width). After the tumor volume of mice approached about 100 mm^3, these mice were randomly grouped and injected with PBS or **faMOF**. The dose of **faMOF** was established based on the studies on the curative effect and safety of 6-TG (20 mg/day).[S2,S3] Based on the principle of surface area normalization and other iodine-containing nanomaterials used in radiotherapy,[S4-S6] the dosage of 1.0 mg/kg was adopted. The tumor-bearing mice (n = 5 mice per group) were intratumorly injected with different formulations (1 mg/kg, every other day). For PBS + X-ray and **faMOF** + X-ray groups, mice were exposure to X-ray (4 Gy) every three days. All injections were administered in total, at 2-day intervals. Tumor volumes were measured every day for 14 days. Finally, the mice were humanely killed and the tumors were harvested after 14 d of treatment.

H&E and immunohistochemical staining. The mice were killed after 14 d of treatment, and representative heart, liver, spleen, lung, kidney, and tumor tissues were collected for histology analysis. The tissues were sliced and dehydrated, dehydration and stained with haematoxylin and eosin (H&E). The H&E staining images were observed under the brightfield microscopy (Olympus, Japan). For immunohistochemistry assays, tumors slices were treated for immunohistochemistry, according to the manufacturer's instructions.

Supporting Figures

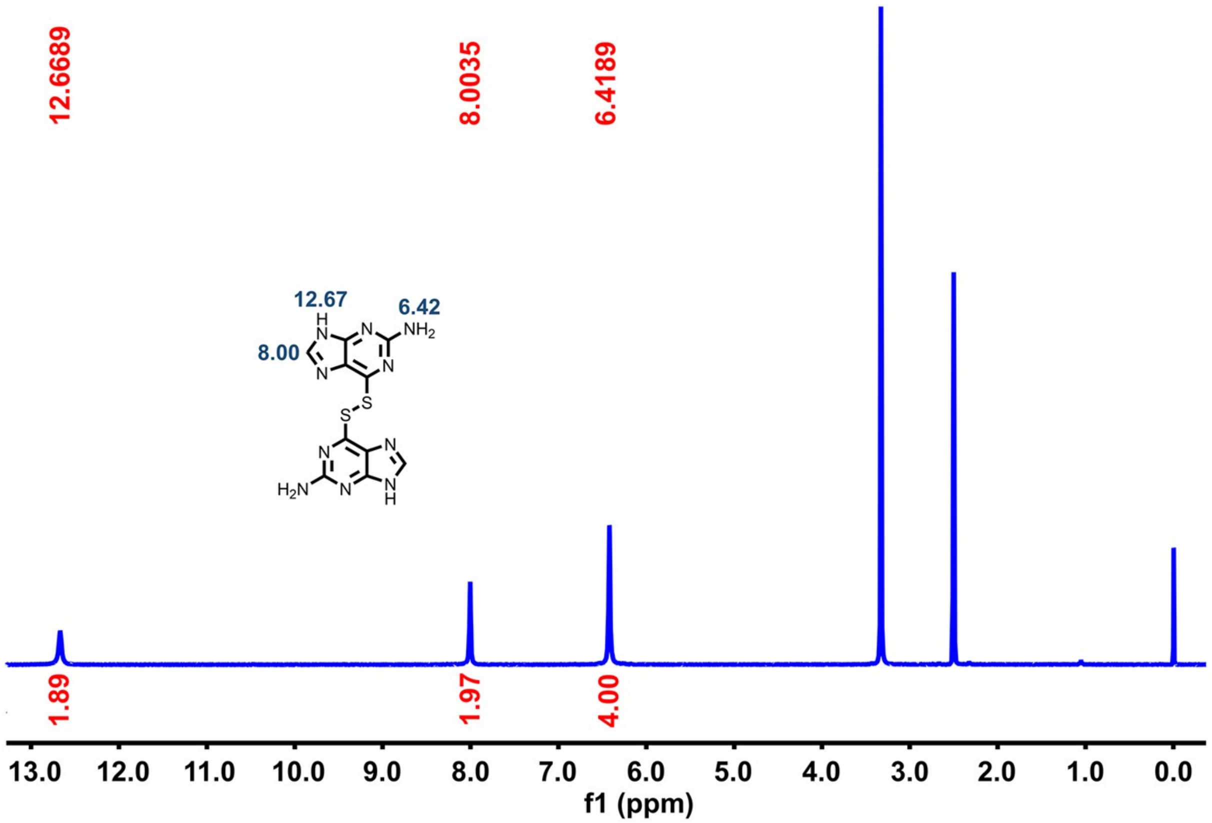

Fig. S1. ^{1}H NMR spectrum of TGSSTG in DMSO-d_6 at 400 MHz.

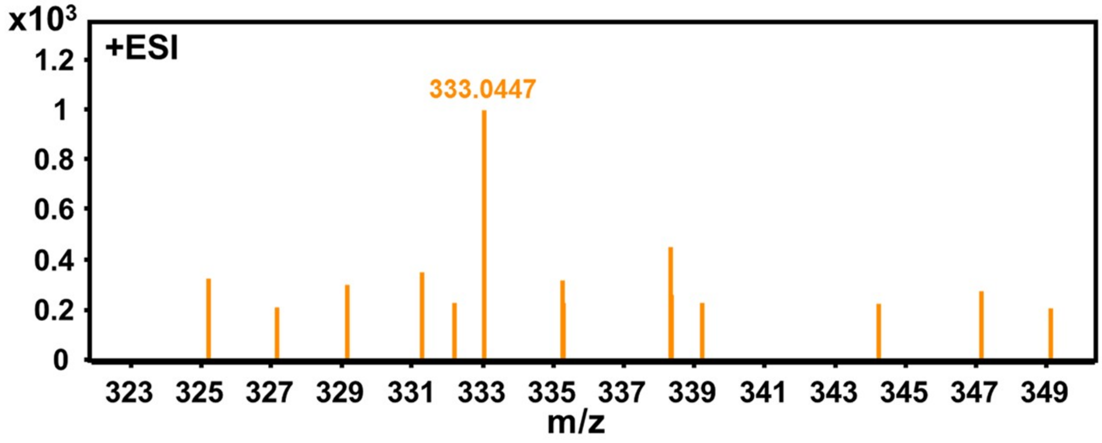

Fig. S2. Mass spectrum analysis of TGSSTG. HRESIMS m/z 333.0447 $[M+H]^+$ (calcd for $C_{10}H_9N_{10}S_2$, 333.0448).

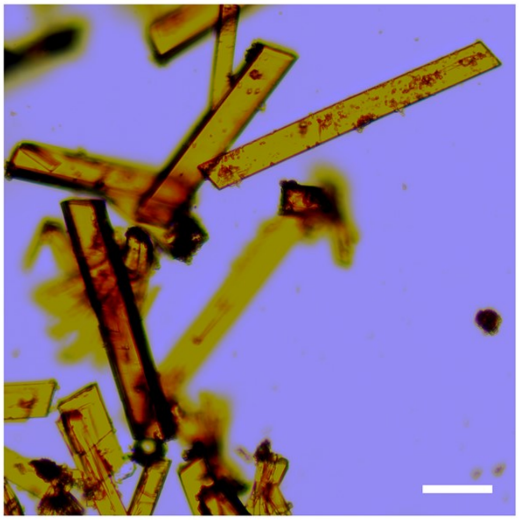

Fig. S3. Crystal image of **aaMOF**, scale bar = 100 μm.

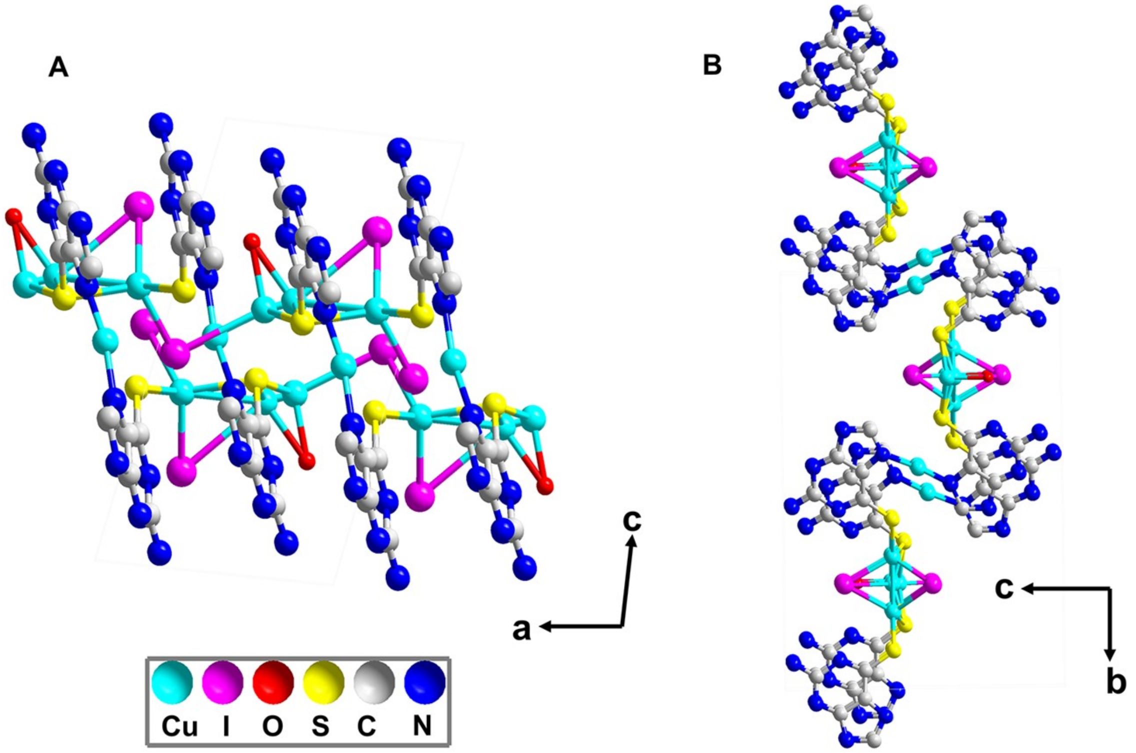

Fig. S4. Structure of **aaMOF** along (A) the *b*-axis and (B) *a*-axis.

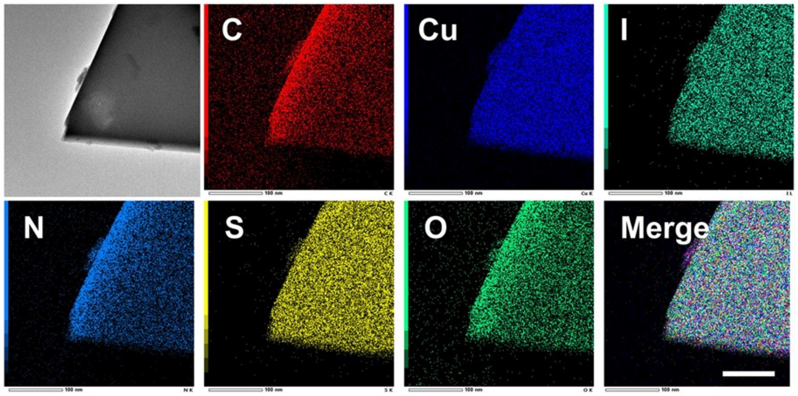

Fig. S5. Elemental mapping of **aaMOF** (scale bar = 100 nm).

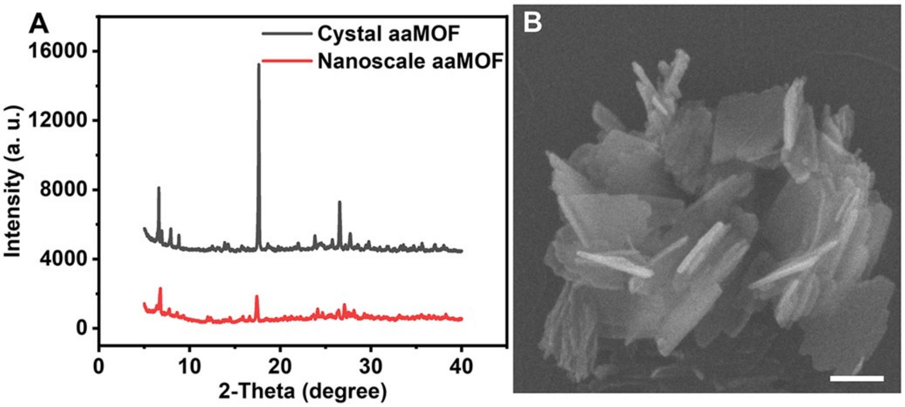

Fig. S6. (A) PXRD and (B) scanning electron microscope image of nanoscale **aaMOF**. Scale bar = 200 nm.

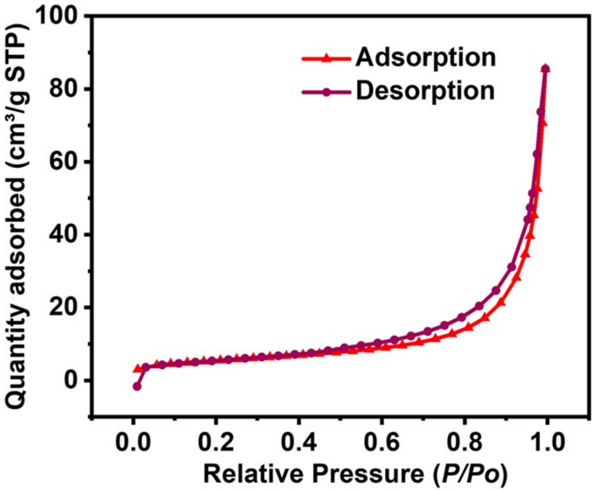

Fig. S7. The N_2 adsorption/desorption isotherms of **aaMOF**.

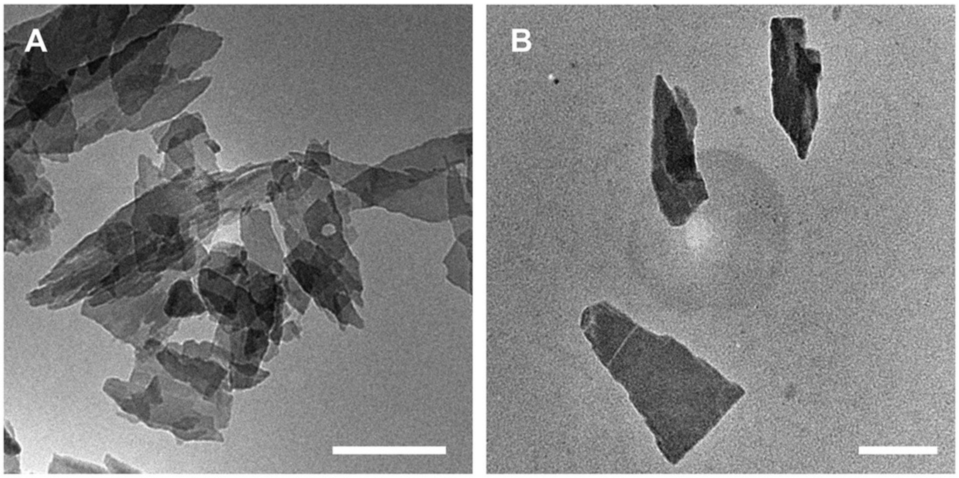

Fig. S8. TEM images of nanoscale (A) **aaMOF** and (B) **faMOF**, scale bars = 100 nm.

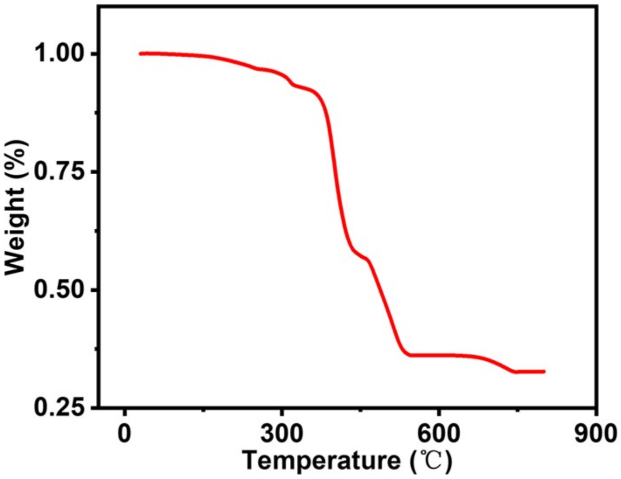

Fig. S9. The thermogravimetry analysis in air atmosphere of **aaMOF**.

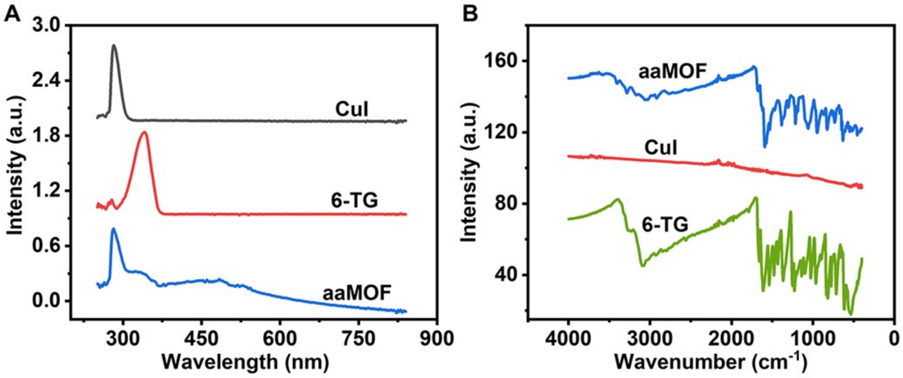

Fig. S10. (A) UV-vis and (B) infrared spectra of CuI, 6-TG, and **aaMOF**.

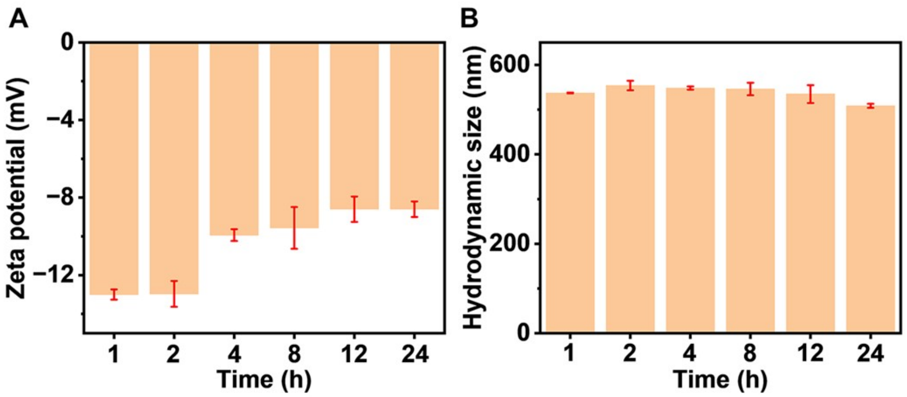

Fig. S11. (A) Zeta potential and (B) hydrodynamic size distribution of **faMOF** over time in PBS (pH = 7.4).

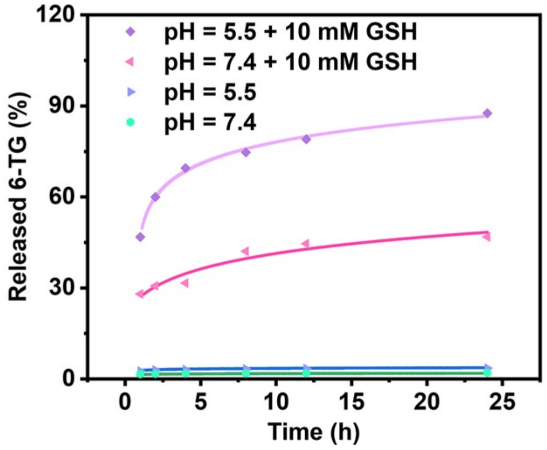

Fig. S12. Cumulative release and the dynamic fitting curves of **aaMOF** under different conditions.

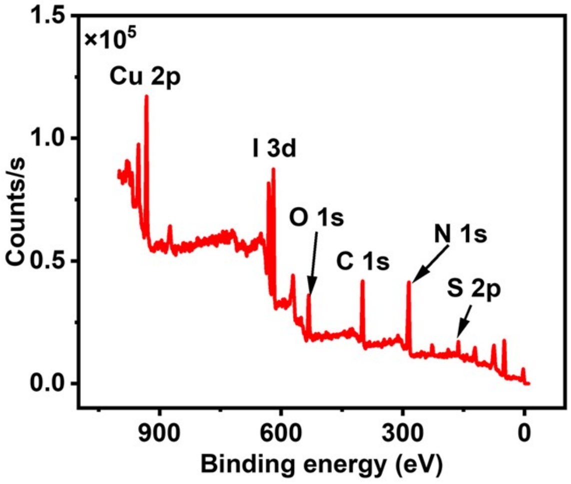

Fig. S13. XPS survey spectrum of **aaMOF**.

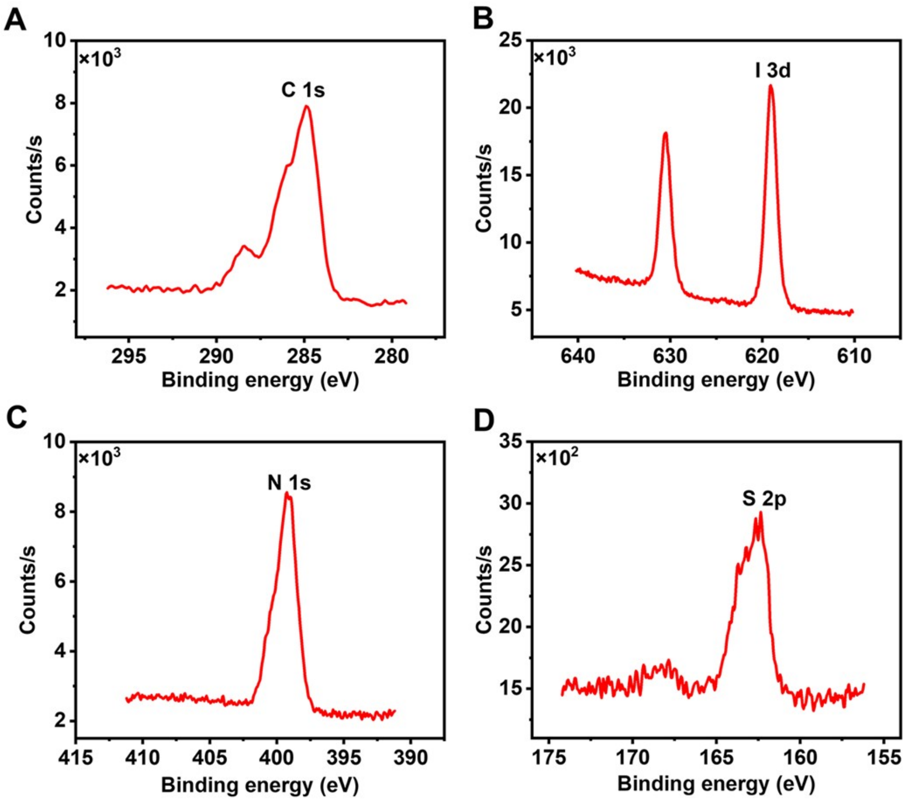

Fig. S14. High resolution XPS spectra of **aaMOF** for (A) C 1s, (B) I 3d, (C) N 1s, and (D) S 2p.

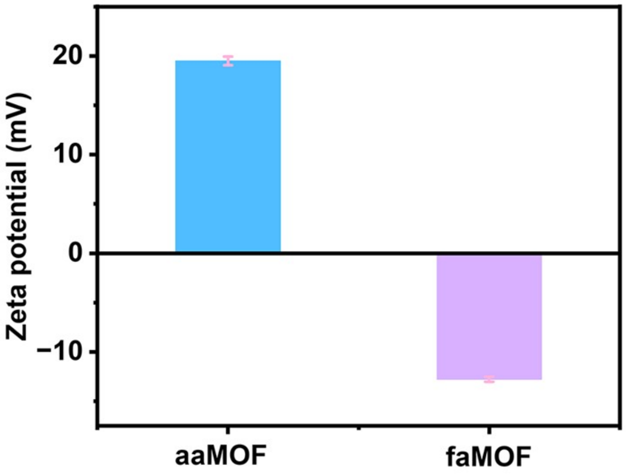

Fig. S15. Zeta potentials of **aaMOF** and **faMOF**.

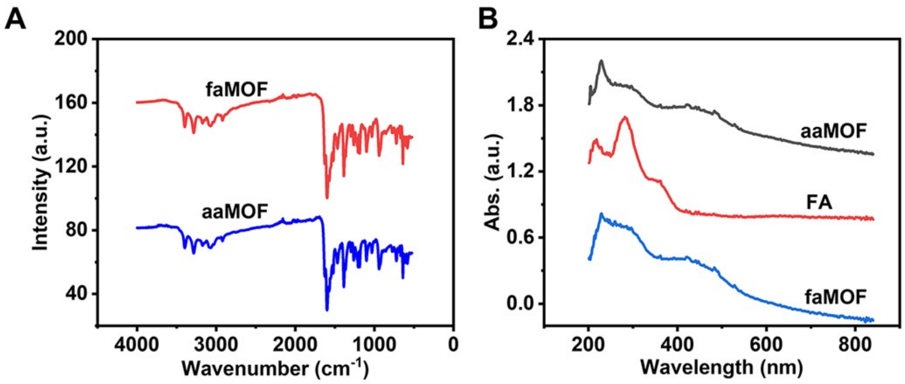

Fig. S16. (A) Infrared and (B) UV-vis spectra of **aaMOF**, FA, and **faMOF**.

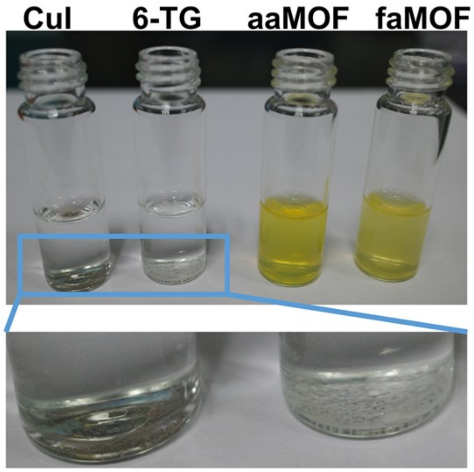

Fig. S17. Photographs of aqueous dispersion of CuI, 6-TG, **aaMOF**, and **faMOF**.

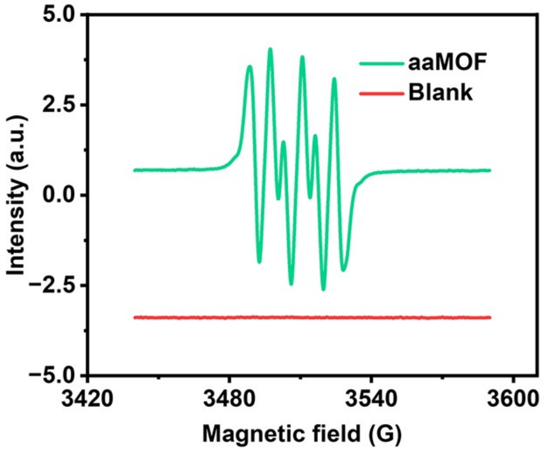

Fig. S18. EPR signals of $^{\bullet}O_2^{-}$ using DMPO as a radical scavenger in the presence of **aaMOF** and 8 mM H_2O_2.

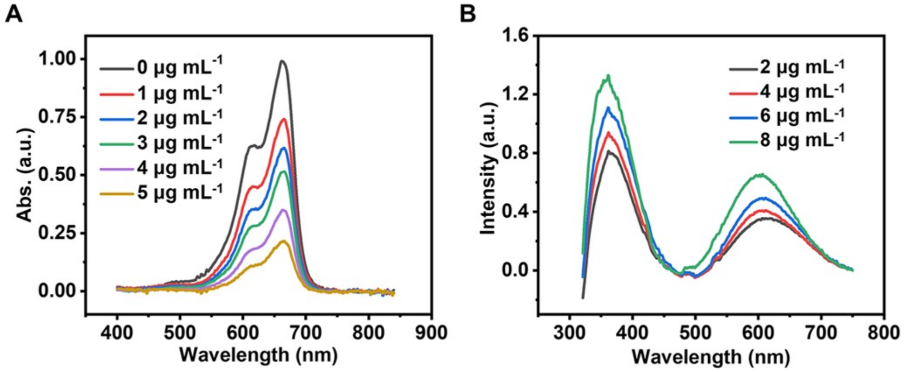

Fig. S19. UV-vis of (A) MB and (B) TMB after incubation with various concentrations of **aaMOF** and 8 mM H_2O_2 in aqueous solution.

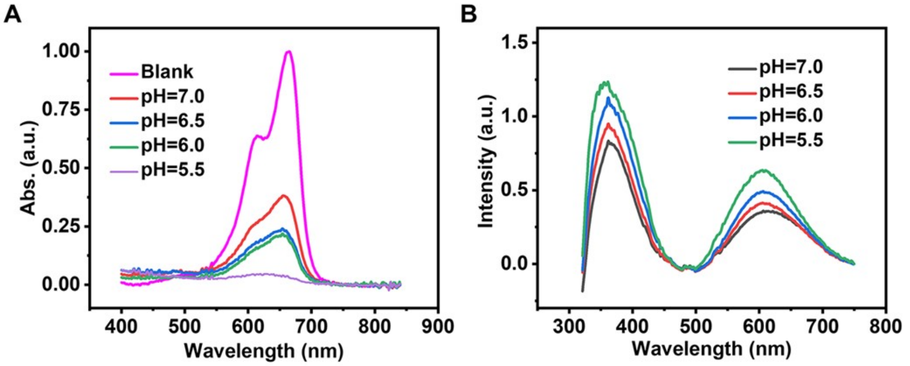

Fig. S20. UV-vis of (A) MB and (B) TMB after incubation with **aaMOF** and 8 mM H_2O_2 in aqueous solution at various pHs.

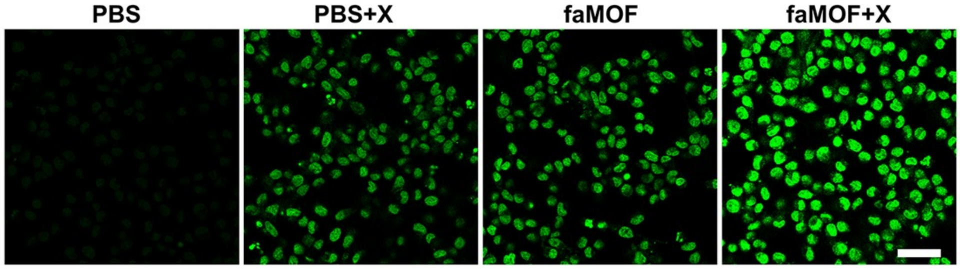

Fig. S21. Confocal laser scanning micrographs (CLSM) images of cells stained with DCFH-DA after treated with PBS, PBS + X-ray, **faMOF**, and **faMOF** + X-ray, scale bar = 50 μm.

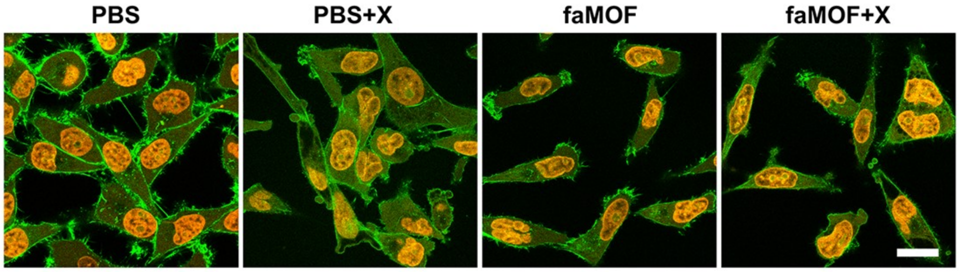

Fig. S22. CLSM images of cells stained with Hoechst 33342 and Rhodamine-labelled Phalloidin after treated with PBS, PBS + X-ray, **faMOF**, and **faMOF** + X-ray, scale bar = 20 μm.

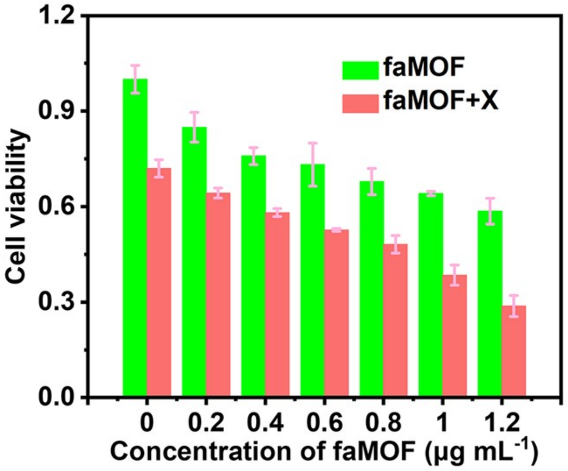

Fig. S23. Cell viabilities of cells treated with various concentrations of **faMOF** under X-ray radiation.

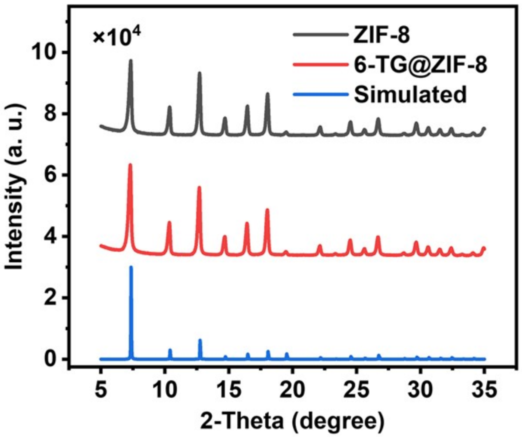

Fig. S24. PXRD patterns of experimental ZIF-8, 6-TG@ZIF-8, and simulated ZIF-8.

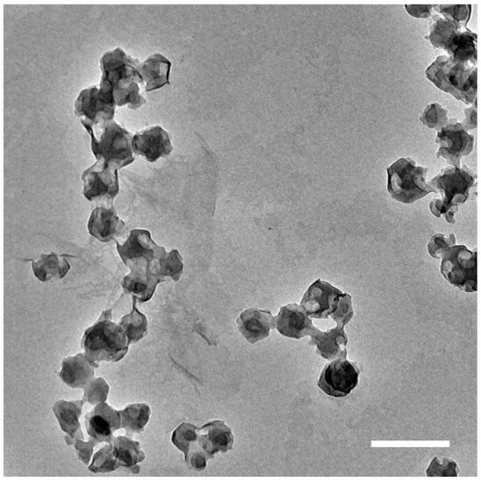

Fig. S25. TEM image of synthesized 6-TG@ZIF-8, scale bar = 200 nm.

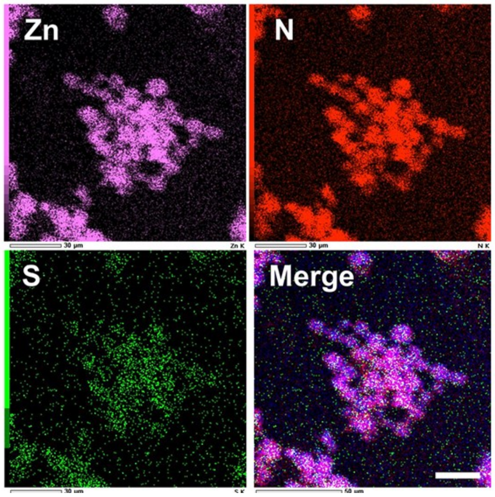

Fig. S26. Elemental mapping of synthesized 6-TG@ZIF-8, scale bar = 200 nm.

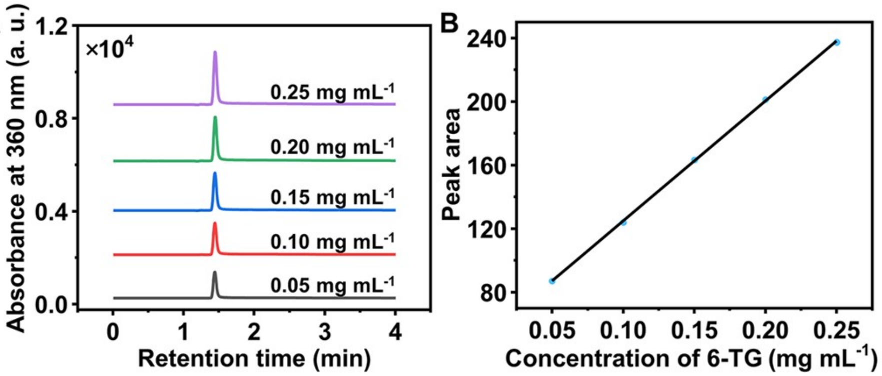

Fig. S27. (A) Ultra-performance liquid chromatography analysis of 6-TG with different concentrations. (B) Standard curve of 6-TG concentrations Vs peak area of UV absorbance at 330 nm.

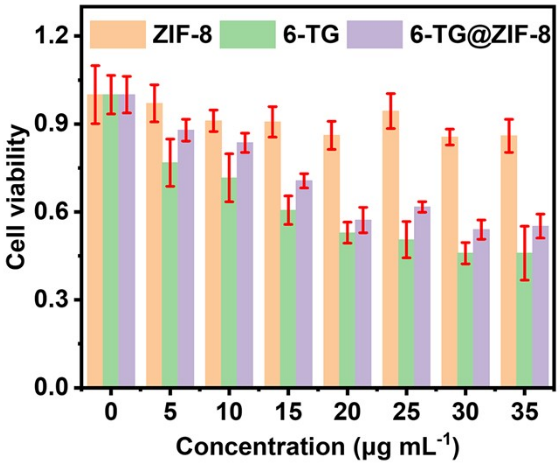

Fig. S28. Cell viabilities of cells treated with various concentrations of ZIF-8, 6-TG, and 6-TG@ZIF-8.

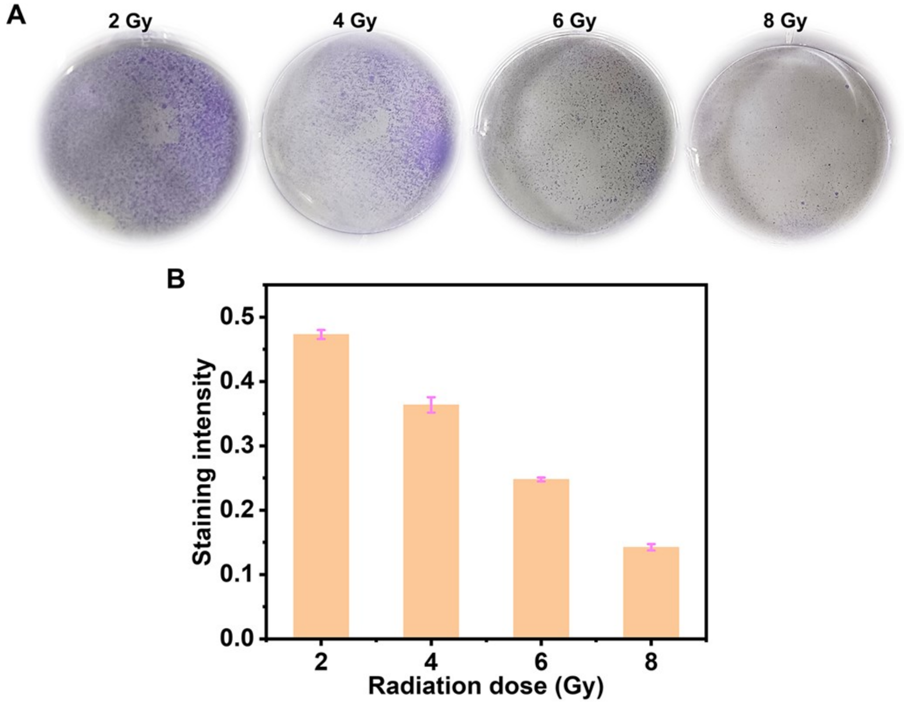

Fig. S29. (A) Clonogenic assay of cells treated with **faMOF** and various doses of X-ray, cells were stained with crystal violet. (B) Staining intensity of crystal violet extracted from colonies received different radiation doses.

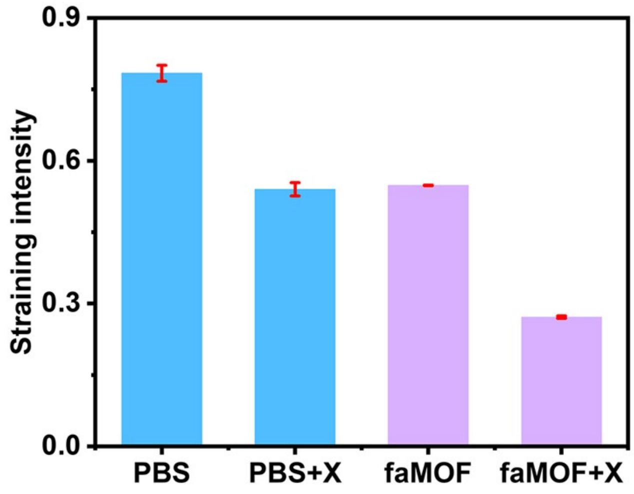

Fig. S30. Staining intentity of crystal violet extracted from colonies after cells were treated with PBS, PBS + X-ray, **faMOF**, and **faMOF** + X-ray.

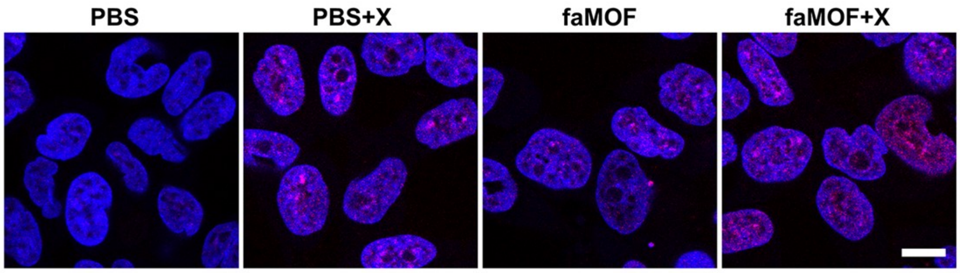

Fig. S31. Reprehensive CLSM images of immunofluorescence against PARP, cells were treated with PBS, PBS + X-ray, **faMOF**, and **faMOF** + X-ray, and stained with Hoechst 33342. Scale bar = 10 μm.

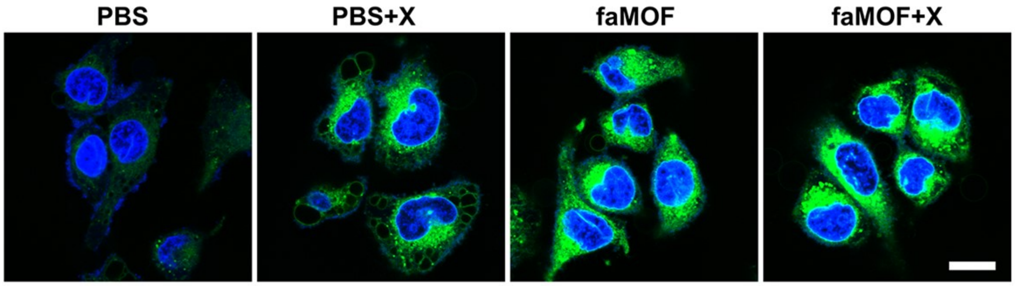

Fig. S32. CLSM images of cells treated with PBS, PBS + X-ray, **faMOF**, and **faMOF** + X-ray, cells were stained with Hoechst 33342 and BODIPY C11, scale bar = 10 μm.

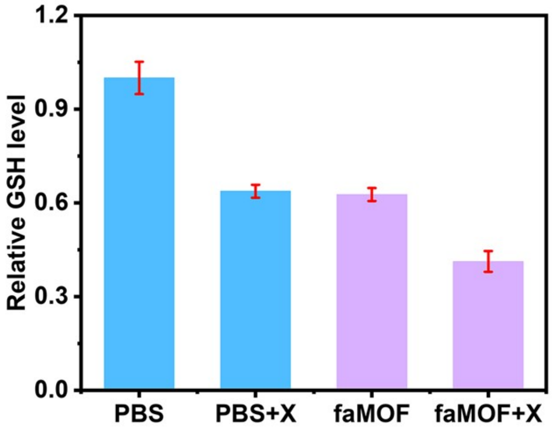

Fig. S33. Relative GSH levels in PBS, PBS + X-ray, **faMOF**, and **faMOF** + X-ray treated cells.

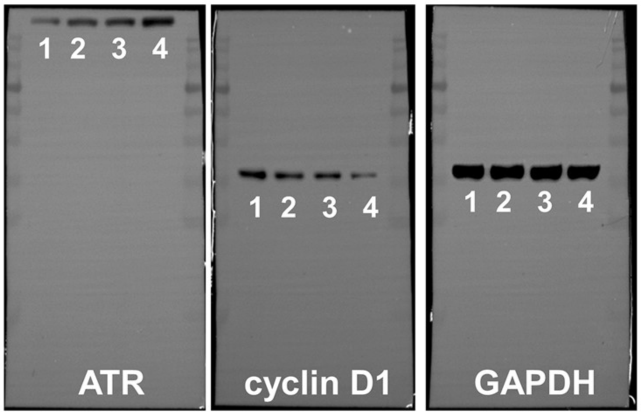

Fig. S34. Western blot analysis of ATR and cyclin D1, cells were treated with PBS (1), PBS + X-ray (2), **faMOF** (3), and **faMOF** + X-ray (4).

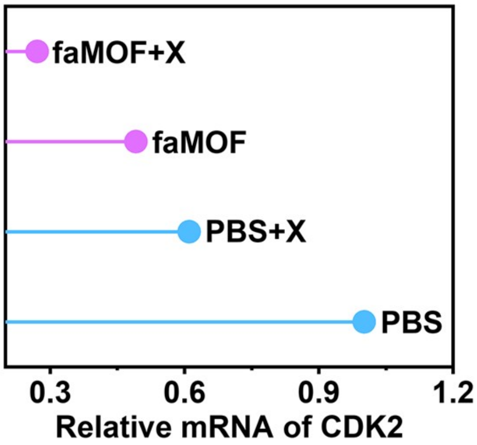

Fig. S35. Relative CDK2 mRNA level in cells treated with PBS, PBS + X-ray, **faMOF**, and **faMOF** + X-ray.

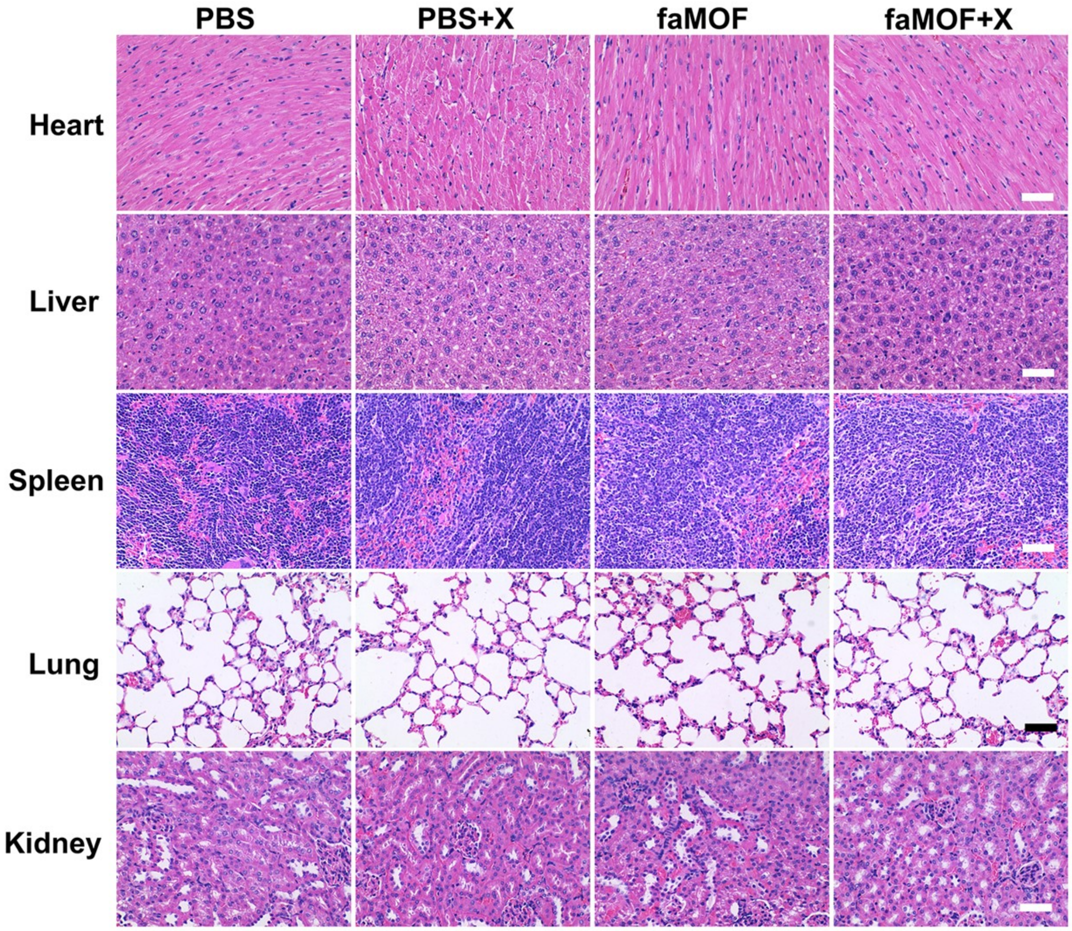

Fig. S36. H&E stained organ slices taken after 14 day-treatment (scale bar = 50 μm).

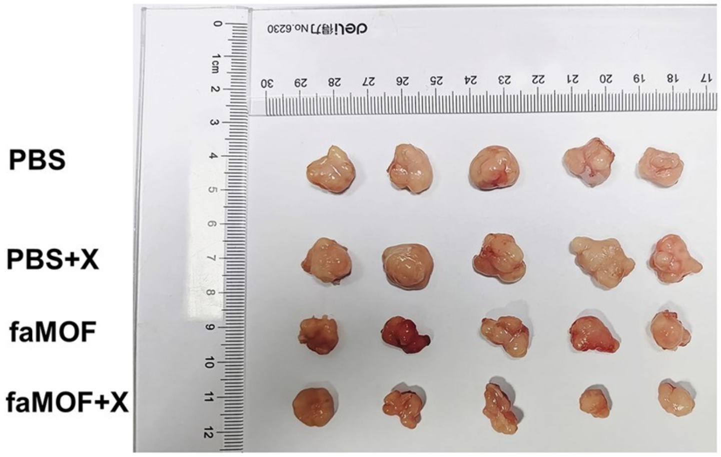

Fig. S37. Tumors extracted from mice at the end of 14 days' treatment in each group.

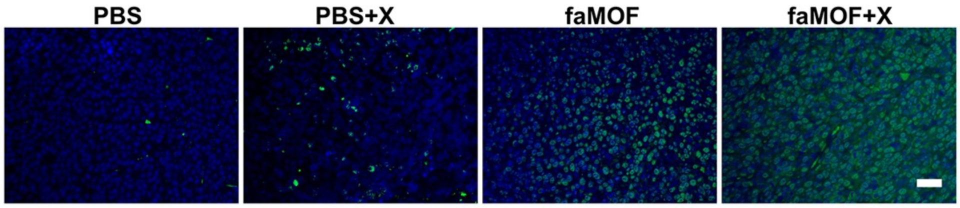

Fig. S38. Immunofluorescence staining against PARP of tumor tissues after various treatments, scale bar = 20 μm.

Reference

[S1] A. J. van der Vlies, U. Hasegawa and J. A. Hubbell, *Mol. Pharmaceutics*, 2012, **9**, 2812-2818.

[S2] A. Jena, P. B. Neelam, H. Telaprolu, U. K. Mangipudi, U. Dutta, S. Sebastian and V. Sharma, *Clin. Res. Hepatol. Gastroenterol.*, 2023, **47**, 102155.

[S3] J.-B. Chevaux and L. Peyrin-Biroulet, *Dig. Liver Dis.*, 2011, **43**, 96-97.

[S4] S. Reagan-Shaw, M. Nihal and N. Ahmad, *The FASEB Journal*, 2008, **22**, 659-661.

[S5] M. Yin, Y. Yuan, Y. Huang, X. Liu, F. Meng, L. Luo, S. Tian and B. Liu, *ACS Nano*, 2024, **18**, 8325-8336.

[S6] B. L. Cline, W. Jiang, C. Lee, Z. Cao, X. Yang, S. Zhan, H. Chong, T. Zhang, Z. Han, X. Wu, L. Yao, H. Wang, W. Zhang, Z. Li and J. Xie, *ACS Nano*, 2021, **15**, 17401-17411.
